# Analysis of cell-based RNAi screens

**DOI:** 10.1186/gb-2006-7-7-r66

**Published:** 2006-07-25

**Authors:** Michael Boutros, Lígia P Brás, Wolfgang Huber

**Affiliations:** 1 Signaling and Functional Genomics, German Cancer Research Center, Im Neuenheimer Feld 580, 69120 Heidelberg, Germany; 2 EMBL - European Bioinformatics Institute, Cambridge CB10 1SD, UK; 3 Centre for Chemical and Biological Engineering, IST, Technical University of Lisbon, Av. Rovisco Pais, P-1049-001 Lisbon, Portugal

## Abstract

RNA interference (RNAi) screening is a powerful technology for functional characterization of biological pathways. Interpretation of RNAi screens requires computational and statistical analysis techniques. We describe a method that integrates all steps to generate a scored phenotype list from raw data. It is implemented in an open-source Bioconductor/R package, *cellHTS * (). The method is useful for the analysis and documentation of individual RNAi screens. Moreover, it is a prerequisite for the integration of multiple experiments.

## Abstract 2

cellHTS is a new method for the analysis and documentation of RNAi screens.

## Rationale

RNA interference (RNAi) is a conserved biological mechanism to silence gene expression on the level of individual transcripts. RNAi was discovered in *Caenorhabditis elegans *when Fire and Mello [1] observed that injecting long double-stranded (ds) RNAs into worms led to efficient silencing of homologous endogenous RNAs. Subsequent studies showed that the RNAi pathway is conserved in *Drosophila *and vertebrates, and can be used as a tool to downregulate the expression of genes in a sequence specific manner [2,3]. Long dsRNAs are commonly used in *Drosophila *and *C. elegans*. In mammalian cells, long dsRNAs induce an interferon response, and therefore short 21 mer RNA duplexes (small interfering RNAs [siRNAs]) are effective in silencing target mRNAs [4,5].

**Figure 1:**
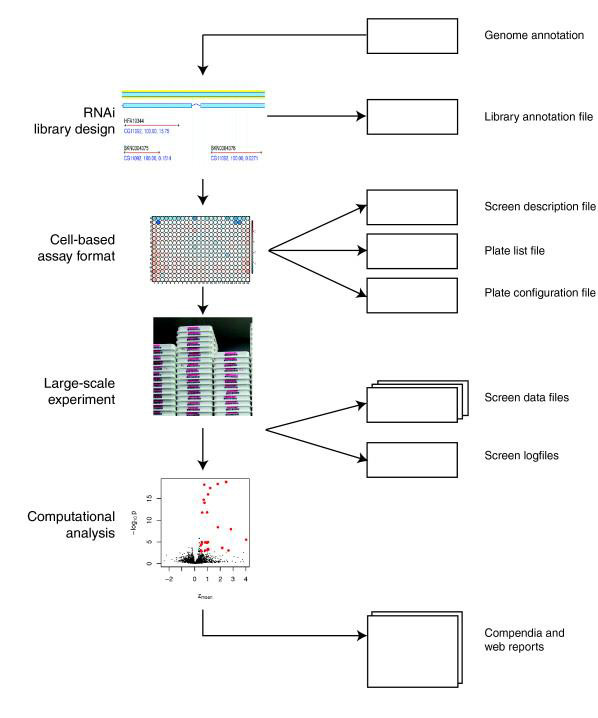
Experimental steps in a cell-based HTS assay. A cell-based HTS assay consists of a set of experimental steps, shown in the left part of the figure, which are recorded in a set of corresponding data structures, shown in the right part of the figure. HTS, high-throughput screening.

Cell-based RNAi screens open new avenues for the systematic analysis of genomes. Traditionally, genetic screens by random mutagenesis have been successful in identifying and characterizing genes in model organisms that are required for specific biological processes [6]. These led to the discovery of many pathways that were later implicated in human disease. However, the identification of genes whose mutation leads to an altered phenotype can be cumbersome and slow. Rapid reverse genetics by RNAi allows the systematic screening of a whole genome whereby every single transcript is depleted by siRNAs or dsRNAs. Genes with unknown functions can then be classified according to their phenotype. The speed of reverse genetic screens using high-throughput technologies promises to accelerate significantly the functional characterization of genes [7]. RNAi screens have been successfully used in *C. elegans *to elucidate whole organism phenotypes and for cell-based assays in fly, mouse, and human cells [8-17]. Figure 1 outlines the main steps in cell-based high-throughput screening (HTS) experiments.

The analysis of data sets generated by high-throughput phenotypic screens poses new methodological challenges. The richness of phenotypic results can range from single numerical values to multidimensional images from automated microscopy. Whereas analysis of functional genomic datasets generated by transcriptome and proteome analysis has attracted considerable interest, analysis of high-throughput cell-based assays has lagged behind. Each study has been conducted using unique custom-tailored analytical methods. Although this may be appropriate within the context of a single study, it makes the integration or comparison of datasets difficult if not impossible. The documentation and minimal information required for reporting RNAi experiments remain unresolved issues [18]. Nevertheless, as the number of RNAi screens performed by different groups increases, it will be instrumental that reliable tools are developed for their integration and comparative analysis.

**Figure 2:**
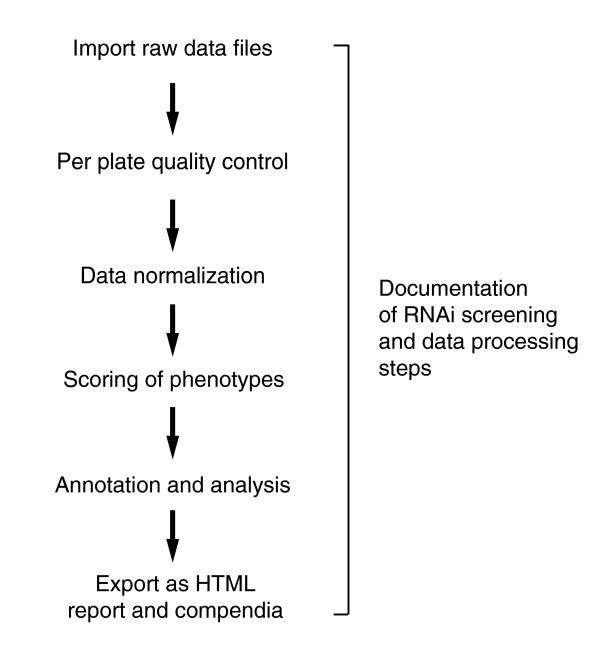
Analysis steps for a cell-based HTS assay. The main steps in the computational analysis of a cell-based HTS assay. HTS, high-throughput screening.

We present a software package for the construction of analysis pipelines for genome-wide RNAi screens. Step by step, it leads from raw data files to annotated phenotype lists and documentation (Figure 2). Comprehensive data visualization and quality control plots aid in identifying experimental outliers. The data can be normalized for systematic technical variations, and statistical summaries are calculated. Quality metrics of the experiment help in assessing the strength of the results. The complete analysis is documented as a computer-readable living document. A navigable presentation of the results is produced as a set of HTML pages that is amenable, for example, for provision as supplemental information alongside publication of the study.

## Example data

We demonstrate the analysis methodology using a published example dataset from a genome-wide RNAi screen for dsRNAs that cause cell viability defects in cultured *Drosophila *cells [9]. In these experiments, Kc_167 _cells were treated with dsRNAs from a library consisting of more than 20,000 dsRNAs. After 5 days cell viability was determined using a luminescence readout by a microplate reader. The library was provided in an arrayed format, in which each location in a 96-well or 384-well microplate uniquely identifies the dsRNA. The cell viability screen was performed in duplicate, and raw results are available as plate reader outputs containing relative luminescence readings. Details of the screening procedure are described elsewhere [9], sequence information is available from our website [19], and the data are provided as part of the examples in the documentation of the *cellHTS *package. The analysis we present here generally follows the analysis performed for the original report [9].

Additionally, we provide a sample dataset of a dual channel experiment. This type of experimental design is used to measure, for instance, the phenotype of a pathway-specific reporter gene against a constitutive reporter that can be used for normalization purposes. Typical examples for such experimental setups are dual-luciferase assays, whereby both firefly and *Renilla *luciferase are measured in the same well. In principle, multiplex assays can consist of many more than two channels, such as in the case of flow-cytometry readout [20] or other microscopy-based high-content approaches.

## Data import and assembly

In this section we discuss the information that is necessary to describe a cell-based HTS experiment. In addition to the primary data files, descriptions of the experimental setup, the configuration of screening plates, and annotations for the RNAs need to be provided. A schematic representation of a screening setup and the corresponding files is shown in Figure 1. The input data consist of several tabular files: the annotation of the library, a *screen description *file, a *plate list *file, a *plate configuration *file, the primary data, and - if available - a log file of the screening procedure.

The *screen description *file contains a general description of the screen, its goal, the conditions under which it was performed, references, and any other information that is important for the analysis and biological interpretation of the experiment. The purpose of this file is similar to that of the experiment design section of a MIAME-compliant dataset [18].

The *plate configuration *file contains information about the common layout of the plates in the experiment, and it assigns each well to one of the following categories: sample (for wells that contain genes of interest), control, empty, and other. This information is used by the software in the normalization, quality control, and gene selection calculations. By default, two types of controls are considered: 'pos' for positive controls and 'neg' for negative controls. Optional parameters allow the definition of further types of controls. Table 1 shows some lines from the *plate configuration *file of the example dataset. Whereas generally the same plate configuration will be used for the whole experiment, a column named batch can be used to define multiple plate configurations.

In the example dataset, the primary data are provided as a set of individual files, one for each replicate measurement per each plate. Each file contains the coordinates for each well and a luminescence value as measured by a plate reader. An example input file is shown in Table 2. When different reporters are employed, there is usually a separate set of files for each reporter.

The names of all primary data files are contained in the *plate list *file, together with their plate identifier, the replicate number, and - if there are several reporters - the identifier name of the reporter. The first lines of the *plate list *file for the example dataset are shown in Table 3.

The *library annotation *file lists the set of RNAi probes in the library together with the identifiers of plates and wells into which they were arrayed. The primary identifier should relate to the molecular entity; for example, it could be the siRNA or dsRNA sequence itself or a unique identifier. In addition, further information can be provided, such as predicted target gene annotation collected from public databases. The first lines of the *library annotation *file for the example data are shown in Table 4.

The *screen log *file can be used to flag individual measurements for exclusion from the analysis. Each row corresponds to one flagged measurement, identified by the filename and the well identifier. The type of flag is specified in the column *Flag*. Most commonly, this will have the value 'NA', indicating that the measurement should be discarded and regarded as missing (for instance, because of contamination). The first few lines of the *screen log *file for the example dataset are shown in Table 5.

Using *cellHTS*, the first processing step is to aggregate all of these files into an R/Bioconductor data object. The files are checked for completeness and correct formatting. Details of the procedure are described in the documentation of the *cellHTS *software.

## Normalization and transformation of the data

### Single channel experiments

**Figure 3:**
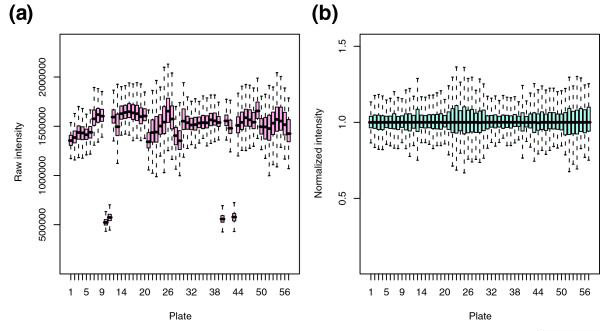
Plate normalization. Box plots of signal intensities in the first replicate set of the example data, grouped by plate. **(a) **Raw data and **(b) **after normalization.

Figure 3a shows box plots of signal intensities in the first replicate set of the example data, grouped by plate. In the experiment the assignment of dsRNAs to plates was quasi-randomized, and so the distribution of signal intensities should not be significantly different between different plates. However, as shown in Figure 3a, the absolute intensity values can vary between plates (for example, when they are read on different days or because of differences in the plate reader settings). Therefore, a more biologically significant measure of the effect is the signal relative to a typical value per plate, such as the plate median. This can be calculated through plate median normalization, which is provided as a function in the *cellHTS *package. Plate median normalization calculates the relative signal of each well compared with the median of the sample wells in the plate:

yki=xkimedianm(xmi)     (1)
 MathType@MTEF@5@5@+=feaafiart1ev1aaatCvAUfeBSjuyZL2yd9gzLbvyNv2Caerbhv2BYDwAHbqedmvETj2BSbqee0evGueE0jxyaibaiKI8=vI8tuQ8FMI8Gi=hEeeu0xXdbba9frFj0=OqFfea0dXdd9vqai=hGuQ8kuc9pgc9s8qqaq=dirpe0xb9q8qiLsFr0=vr0=vr0dc8meaabaqaciGacaGaaeqabaqadeqadaaakeaacaWG5bWaaSbaaSqaaiaadUgacaWGPbaabeaakiabg2da9maalaaabaGaamiEamaaBaaaleaacaWGRbGaamyAaaqabaaakeaadaWfqaqaaiaab2gacaqGLbGaaeizaiaabMgacaqGHbGaaeOBaaWcbaGaamyBaaqabaGcdaqadiqaaiaadIhadaWgaaWcbaGaamyBaiaadMgaaeqaaaGccaGLOaGaayzkaaaaaiaaxMaacaWLjaWaaeWaceaacaaIXaaacaGLOaGaayzkaaaaaa@4943@

Here *x*_*ki *_is the raw intensity for the *k*^th ^well in the *i*^th ^result file, and *y*_*ki *_is its normalized intensity. The median is calculated among the wells annotated as *sample *in plate *i*. Equation 1 is motivated by the measurement model:

*x*_*ki *_= *λ*_*i*_*c*_*ki*_,     (2)

where *c*_*ki *_is a measure of the true biological effect and *λ*_*i *_is a plate-dependent technical gain factor representing, for example, reagent concentrations or instrument settings. The median term in the denominator of Equation 1 is an estimate for *λ*_*i*_. The box plots of the resulting normalized values are shown in Figure 3b.

Generally, the purpose of normalization is to adjust data for unavoidable, unwanted technical variations in the signal while preserving the biologically relevant ones. There could be systematic spatial gradients within the plates, so-called edge effects caused by evaporation in wells during the screening experiment, or systematic differences in reagent concentration caused by pipetting errors. Some of these variations can be adjusted through *post hoc *data normalization, and it is possible to employ additional or alternative normalization methods in a *cellHTS *workflow. Clearly, such variations can be corrected only to a certain extent, and the quality plots described below can also be used to flag those parts of the experiment that need to be repeated.

### Multiple channel experiments

The accuracy and interpretability of screening experiments can often be improved by using multiple independent reporters. For example, one reporter, *R*_1_, could monitor the total number of viable cells in a well, whereas another reporter, *R*_2_, could monitor the activity of a particular pathway. Such experimental setups are typically used in screens for signaling pathway components, where a pathway inducible readout is normalized against a constitutive reporter [8,15,16]. In this way, it becomes possible to distinguish between changes in the readout caused by depletion of specific pathway components versus changes in the overall cell number. An example analysis of the dual channel dataset described above is provided in the vignette 'Analysis of multi-channel cell-based screens' of the *cellHTS *package.

As an example of the analysis of a high-content screening dataset, the vignette 'Feeding the output of a flow cytometry assay into *cellHTS*' of the *prada *package [20] shows how to import the summary scores for each well of a cell-based screen with flow cytometry readout into *cellHTS*.

Further flexibility is provided by the modular, user-extensible design of *cellHTS*. Researchers can add additional functions, for example for normalization, taking advantage of the extensive statistical modeling and visualization capabilities of the R programming language to develop analysis strategies that are adapted to their biological assay and question of interest.

## Quality metrics

The *cellHTS *package generates various visualizations that help in assessing the quality of the data. We calculate numeric summaries and quality metrics on two levels: on the level of individual plates and the complete screen. Quality metrics on the level of individual plates can already be used while the experiment is being performed, for example to identify problematic plates that need to be repeated or to control experimental procedures. Quality assessment of the whole screening experiment helps with the choice of analysis methods and is a necessary prerequisite when data from multiple screens are to be combined into an integrative analysis of phenotype profiles [21,22].

### Per plate quality metrics

**Figure 4:**
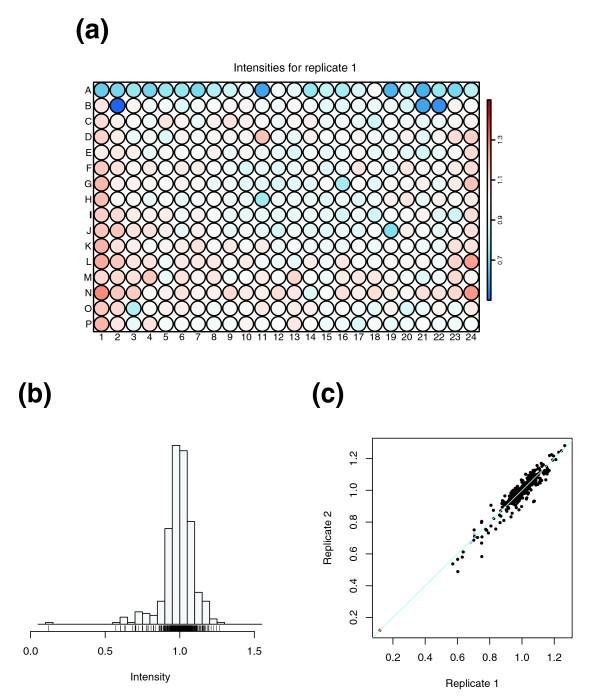
Plate-wise quality plots. **(a) **Plate plot of signal intensities. A false color scale is used to represent the normalized signal. This visualization helps in quickly detecting gross artifacts that manifest themselves in spatial patterns. In the data shown here the values in the top row were consistently low, which could be traced back to a pipetting problem. **(b) **Histogram of the signal intensities. **(c) **Scatterplot between two replicate plate results. Ideally, all points lie on the identity line (x = y).

Figure 4 shows three plots that we produce for every 384-well plate. Figure 4a shows a false color representation of the normalized intensities from a single replicate. This visualization allows the user to quickly detect gross artifacts such as pipetting errors. Figure 4b shows the distributions of results from a single plate. The signal distribution of the normalized signal should be approximately the same between replicates as well as between different plates. Usually, one expects to see a single, well defined peak, and this is required by the subsequent analysis. If the histogram shows an unusual shape or has multiple peaks, this can indicate a problem. In addition, the package *cellHTS *reports the dynamic range, calculated as the ratio between the geometric means of the positive and negative controls. Figure 4c shows the scatterplot between two replicate plate results. It allows assessment of the reproducibility of the assay. Ideally, all points should lie on the identity line (*x *= *y*), and large deviations indicate outliers. There are different ways to quantify the spread of the data around the *x *= *y *line. The package *cellHTS *reports the Spearman rank correlation coefficient; for the data shown in Figure 4c, the correlation coefficient is 0.91.

There are various kinds of experimental artifacts that can be observed at this stage, such as pipetting errors, evaporation of liquid in wells (edge effects), and contamination. Depending on the quality of the data, the screening of individual plates may be repeated; alternatively, individual well positions that appear to be outliers may be flagged for exclusion from subsequent analysis.

### Experiment wide quality metrics

**Figure 5:**
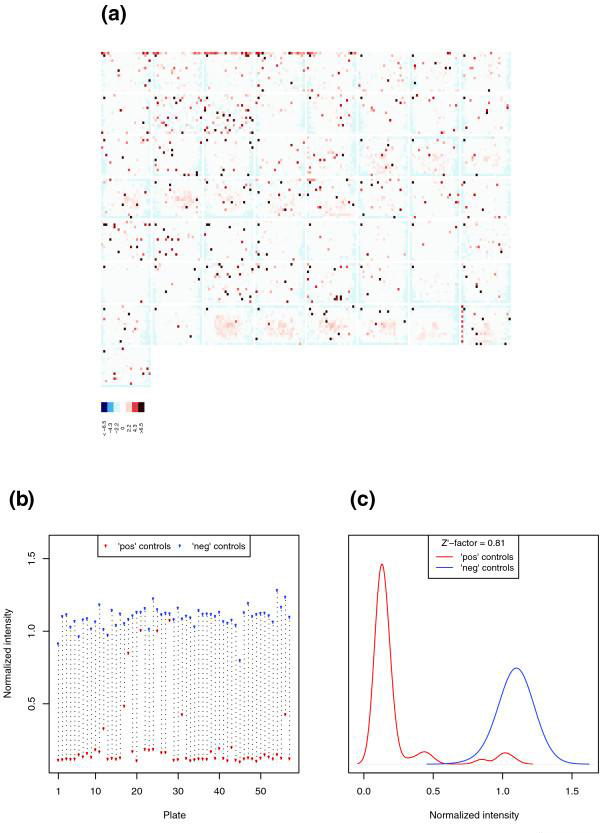
Experiment-wide quality plots. **(a) **Overview of the complete set of *z*-score values from a genome-wide screen of 21,306 dsRNAs. The dsRNAs were contained in 57 plates, laid out in eight rows and eight columns, and the 384 *z*-score values within each plate are plotted in a false color representation whose scale is shown at the bottom of the plot. **(b) **Signal from positive (red dots) and negative (blue dots) controls (y axis) plotted against the plate number (x axis). (c) Distribution of the signal from positive (red line) and negative (blue line) controls, obtained from kernel density estimates. The distance between the two distributions is quantified by the *Z' *factor. ds, double-stranded.

Figures 3 and 5 show four types of plots that are useful in analyzing the experiment's overall quality. When the dsRNAs are randomized between plates and experiments are performed under identical conditions, the box plots of raw data (Figure 3a) should show approximately the same location and scale. Variations can occur, for example when experiments were performed using different batches of reagents. In the example dataset, four of the 384-well plates shown in Figure 3a have much lower median intensities than the others. To an extent, such deviations can be adjusted by normalization, and the box plots for the plate median normalized data are shown in Figure 3b. Calculated statistical parameters, such as dynamic range, can be used to judge whether individual plates need to be repeated.

Figure 5a shows a screen image plot of the *z*-scores (see next section, below) for the more than 20,000 measurements in the experiment. Strong red colors correspond to a large positive *z*-score, which in this experiment is indicative of reduced cell viability. The screen overview can highlight problematic measurements, for example a row of relatively low measurements (indicated in red), which might have been caused by the same pipetting or plate reader artifact that was already indicated by Figure 4a. These wells can be flagged and excluded from the analysis.

Figures 5b and 5c look specifically at the controls. For each plate, Figure 5b shows the normalized intensities from positive (red dots) and negative (blue dots) controls. Figure 5c shows the distributions of positive and negative control values across plates, represented by density estimates. Whereas the negative controls scatter around 1.1, the positive controls have an average of about 0.1, which indicates a strong cell viability phenotype. A popular parameter in HTS experiments to assess the quality of assays is the ratio of the separation between these two peaks to the assay dynamic range, as measured using the so-called *Z' *factor [23]:

Z′=1−3σpos+σneg|μpos−μneg|,     (3)
 MathType@MTEF@5@5@+=feaafiart1ev1aaatCvAUfeBSjuyZL2yd9gzLbvyNv2Caerbhv2BYDwAHbqedmvETj2BSbqee0evGueE0jxyaibaiKI8=vI8tuQ8FMI8Gi=hEeeu0xXdbba9frFj0=OqFfea0dXdd9vqai=hGuQ8kuc9pgc9s8qqaq=dirpe0xb9q8qiLsFr0=vr0=vr0dc8meaabaqaciGacaGaaeqabaqadeqadaaakeaaceWGAbGbauaacqGH9aqpcaaIXaGaeyOeI0IaaG4mamaalaaabaGaeq4Wdm3aaSbaaSqaaiaabchacaqGVbGaae4CaaqabaGccqGHRaWkcqaHdpWCdaWgaaWcbaGaaeOBaiaabwgacaqGNbaabeaaaOqaamaaemGabaGaeqiVd02aaSbaaSqaaiaabchacaqGVbGaae4CaaqabaGccqGHsislcqaH8oqBdaWgaaWcbaGaaeOBaiaabwgacaqGNbaabeaaaOGaay5bSlaawIa7aaaacaGGSaGaaCzcaiaaxMaadaqadiqaaiaaiodaaiaawIcacaGLPaaaaaa@53C8@

where *μ*_pos _and *μ*_neg _are the mean values of positive and negative controls, and *σ*_pos _and *σ*_neg _are their standard deviations. For Normal distributed data, the expression (*σ*_pos_^2 ^+ *σ*_neg_^2^)^1/2 ^would be more natural than *σ*_*pos *_+ *σ*_neg _in the numerator, but the definition given in Equation 3 is what has been used in the literature and in practice. In the *cellHTS *software, we use robust estimators for μ and σ. *Z' *is dimensionless and is always 1 or less. The obtained values can be used as a rough estimate of the quality of the cell-based assay. Zhang and coworkers [23] gave the following classification: *Z' *= 1, an optimal assay; 1 > *Z' *≥ 0.5, an excellent assay that allows quantitative distinction of obtained phenotypes; 0.5 > *Z' *> 0, an assay with limited quantitative information; and *Z' *≈ 0, a 'yes/no' type assay. Although this categorization certainly depends on the choice of positive and negative controls, it can provide guidance when designing cell-based assays. The sample dataset, for example, had a calculated *Z' *factor of 0.81.

## Scoring and identification of candidate modifiers

As a next step in the analysis, phenotypes must be scored for their statistical significance. This step calculates a single number, a score, for each dsRNA as a measure of evidence for a generated phenotype. Furthermore, a list of top scoring dsRNAs can be selected as the 'hit list' of the screen.

As a first step, we transform the normalized measurements into *z*-scores:

zkj=±ykj−MS,     (4)
 MathType@MTEF@5@5@+=feaafiart1ev1aaatCvAUfeBSjuyZL2yd9gzLbvyNv2Caerbhv2BYDwAHbqedmvETj2BSbqee0evGueE0jxyaibaiKI8=vI8tuQ8FMI8Gi=hEeeu0xXdbba9frFj0=OqFfea0dXdd9vqai=hGuQ8kuc9pgc9s8qqaq=dirpe0xb9q8qiLsFr0=vr0=vr0dc8meaabaqaciGacaGaaeqabaqadeqadaaakeaacaWG6bWaaSbaaSqaaiaadUgacaWGQbaabeaakiabg2da9iabgglaXoaalaaabaGaamyEamaaBaaaleaacaWGRbGaamOAaaqabaGccqGHsislcaWGnbaabaGaam4uaaaacaGGSaGaaCzcaiaaxMaadaqadiqaaiaaisdaaiaawIcacaGLPaaaaaa@432C@

where y_kj _is the normalized value for the *k*^th ^well in the *j*^th ^replicate, and *M *and *S *are mean and standard deviation of the distribution of the y values. In the *cellHTS *software we use the robust estimators median and median absolute deviation to estimate M and S. The choice of the sign (±) in Equation 4 depends on the type of the assay. We want a strong effect to be represented by a large positive *z*-score. For an inhibitor assay, such as in the example data, a strong effect is indicated by small values of *y*_*kj*_, and hence we use a minus sign in Equation 4. For an activator assay, for which a strong effect is indicated by large values of *y*_*kj*_, we would use the plus sign.

To aggregate the values from the replicate experiments into a single number per well, there are different options, and the choice depends on the number of replicates available and the type of follow-up analysis. The least stringent criterion is to take the maximum of the *z*-scores from the replicates; the most stringent one is the minimum and another option is the root mean square.

## Gene annotation

The Bioconductor project, into which the *cellHTS *package is integrated, offers a variety of methods to associate the dsRNAs used in the screen with the annotations of their target genes and transcripts from public databases and with other genomic datasets. These annotations can then be mined for interesting patterns. Many of the methods that were initially developed for gene expression microarrays can be adapted directly. Two basic approaches for the integration of gene annotation data are provided by Bioconductor: downloadable, versioned annotation packages that reside on the user's computer; and clients to public bioinformatics web services, such as provided by the EBI [24].

For the example dataset, the vignette 'End-to-end analysis of cell-based screens: from raw intensity readings to the annotated hit list' of the *cellHTS *package demonstrates how to obtain a comprehensive set of annotations for the targets of the *Drosophila *RNAi library using the *biomaRt *package [25], which provides an interface from R to the biomart web service [26] of the Ensembl project [24].

## Analysis for enrichment of functional groups

One of the immediate questions after analysis of an RNAi screen is which biological processes are represented by the high scoring genes. More generally, one can consider any type of previously known gene list, which we term a category, and ask whether the genes of a category exhibit particularly extreme phenotype scores.

**Figure 6:**
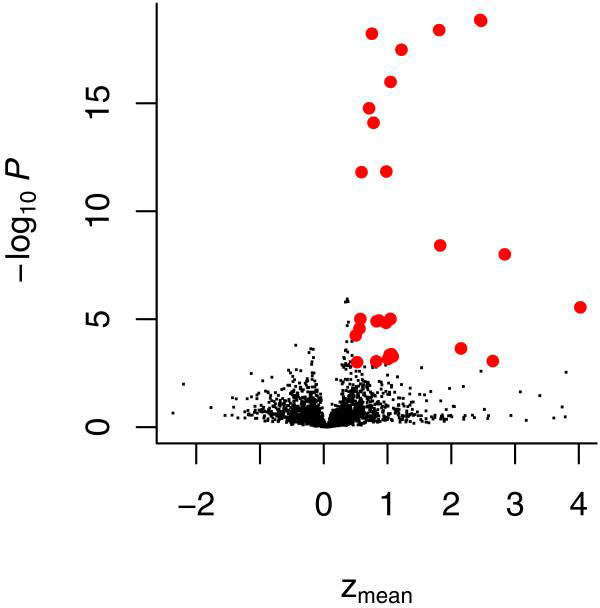
Volcano plot to identify enriched GO categories. Volcano plot of the category analysis. It shows the negative decadic logarithm of the *P *value versus the mean *z*-score for each tested GO category. Categories that are strongly enriched for high-scoring hits are marked in red; details on some of these are shown in Table 6. GO, Gene Ontology.

To search for Gene Ontology (GO) categories [27] that are enriched for high-scoring genes, we employ the *Category *package by Robert Gentleman in Bioconductor. Such an analysis is straightforward; for each possible category of interest, it compares the distribution of scores of genes in the category with the overall distribution. For this comparison, it uses the difference of the means, as well as the statistical significance of the difference as measured by a t-test. The result is shown in Figure 6. Interesting categories are those in the upper right region of the plot; they have both a large difference in means as well as a small *P *value. Table 6 shows selected categories from this plot. In the case of the example dataset, the categories include components of the ribosome (GO:005840; *P *= 2 × 10^-19^) and proteasome (GO:000502; *P *= 1 × 10^-8^). Compared with the original analysis [9], we introduced some technical improvements, such as the use of median and median absolute deviation instead of mean and standard deviation, but for the presented dataset the phenotypic ranking is similar and biological conclusions are the same.

## Reports and living documents

The results of an analysis with the *cellHTS *package are provided in three forms. First, they may be presented as a hyperlinked set of HTML pages that provides access to the input files, all quality-related plots and quality metrics, and the final scored and annotated table of genes. Plots are provided both in PNG and in PDF format. The pages can be browsed with a web browser. We encourage readers to view the example report provided on our website [28].

Second, the *cellHTS *package facilitates the production of a compendium describing the analysis of an RNAi screen. A compendium is a living document that not only reports the result of the computations that were performed to transform a set of input data into an end result, but it also contains the data as well as the human-readable textual description and a machine-readable program of all computations necessary to produce the plots and result tables [29-33]. Readers initially will be presented with a processed document, just like a normal report; however, if they wish they can rerun the analysis, investigate intermediate results, and try variations of the analysis. The *cellHTS *package contains compendia for the analyses of the example data discussed in this report. It uses the vignette and packaging technology available from the R and Bioconductor projects [31,34,35]. All plots shown here are directly taken from the compendium and can be reproduced by users of the package.

Third, the results can be further processed using other software tools. A result with the scores and annotation for all dsRNAs is provided in tabulator delimited text format, which can be imported by spreadsheet programs. Moreover, the complete output of the analysis is stored in a single R object, which can be saved into a file and loaded later for subsequent analysis. The file format is compatible across all operating systems on which R runs.

**Figure 7:**
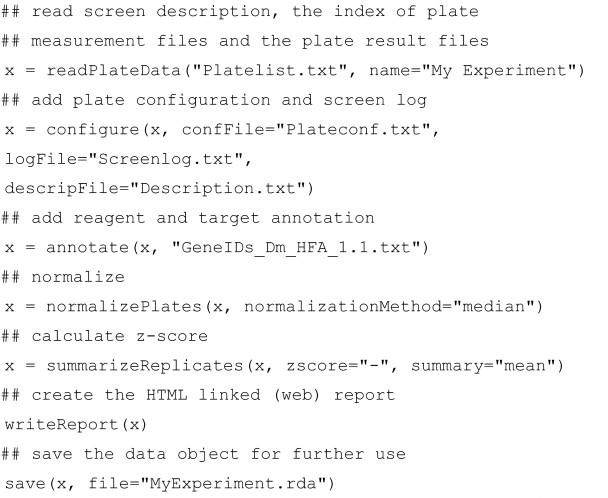
Example *cellHTS *session.

An example session is presented in Figure 7.

A more detailed version with explanation of the input and output of each step and the command options is provided in the documentation of the package *cellHTS*.

## Concluding remarks and outlook

We present a methodology for analysis of cell-based RNAi screens that leads from primary data to a scored and annotated gene list. These steps include data import, normalization for technical variability and quality metrics and plots on the level of individual screening plates and the complete experiment. Results are provided in a hyperlinked HTML report that includes the visualizations, a tabulator delimited scored gene table and a single, comprehensive R data object suitable for subsequent follow-up analyses. The software is available through the free and open source Bioconductor package *cellHTS*.

### Minimal information about RNAi experiments

We have here assumed a working definition of the minimal information about a cell-based RNAi experiment necessary for the analysis. This includes the information in the screen description file and raw instrument readings, as well as information about the plate configuration, which is necessary to visualize spatial effects in phenotype distribution. This is intended as a starting point for discussion; it is certain to be incomplete and will develop with the technology and scientific questions. For example, sequence information on siRNAs or long dsRNAs are necessary to assess potential off-target effects and to annotate the targets when genome annotations change.

There are currently no standard experimental protocols for high-throughput RNAi experiments and, because of rapid developments in RNAi reagents and cell-based assays, we do not expect a limited set of standard protocols to emerge soon. Nevertheless, many of the analysis steps appear to be generic and applicable to many different experiments. Our package is intended to provide tools for creating such an analysis workflow. The analysis functions are customizable, and if needed they can be combined with other functions provided by the user or from other external packages. As the field matures and the community adapts a set of tools that it finds useful, standard analytical methods may emerge [36].

### Specificity and off-target effects of RNAi experiments

The interpretation of large-scale RNAi data relies on annotation of reagents and their specificity. Off-target effects from dsRNAs or siRNAs, which downregulate other transcripts in addition to their intended target, can be caused by relatively short sequence matches. Recent reports have shown that off-target effects can have significant effects on phenotypic readouts. Sequence similarity as small as heptamers with perfect matches in the 3'-untranslated region can mediate translational inhibition of mRNAs through a miRNA pathway [37]. Such effects can have an impact on the annotation of screening results, and phenotypes should be treated with caution until further confirmation can be provided. In addition to improved design algorithms both for dsRNA and siRNA libraries that may minimize off-target effects, a calculated estimate of potential off-target effects could be a useful feature in future releases of *cellHTS *to rank and evaluate scored phenotype lists.

### Outlook

Genome-wide RNAi experiments can be classified as follows: for screens, the goal is the identification of one or few new core components in a specifically assayed process followed by their in-depth genetic and biochemical characterization [17,38]; and for surveys, the aim is the systematic mapping of phenotypic profiles and possibly genetic interaction networks [21,22,39]. Although the individual data points in surveys are rarely independently confirmed and can suffer from higher rates of false negatives and false positives, the fusion of multiple, consistently processed datasets and other large-scale datasets might ultimately provide deeper insights into biological systems [40].

### Software implementation and availability

The package *cellHTS *is available as a freely distributable and open source software package with an Artistic license. It is integrated into the R/Bioconductor [35] environment for statistical computing and bioinformatics, and runs on major operating systems including Windows, Mac OS X, and Unix.

## Additional data files

The following additional data are included with the online version of this article: The R package version 1.3.23 of 5 August 2006 in "source" format (for Unix and Mac OS X; Additional data file 1). The R package in "Windows binary" format (for MS Windows; Additional data file 2). These file archives also contain the example data. A PDF document demonstrating a full end-to-end analysis of the example cell-based screening data (Additional data file 3). A PDF document demonstrating the analysis of multi-channel cell-based screens (Additional data file 4).

## Supplementary Material

Additional data file 1 R package version 1.3.23 of 5 August 2006 in "source" format (for Unix and Mac OS X). This file archive also contains the example data. Click here for file

Additional data file 2 R package in "Windows binary" format. This file archive also contains the example data. Click here for file

Additional data file 3 A PDF document demonstrating a full end-to-end analysis of the example cell-based screening data. Click here for file

Additional data file 4 A PDF document demonstrating the analysis of multi-channel cell-based screens. Click here for file

## Figures and Tables

**Table 1:** Plate configuration file

Batch	Well	Content
1	B01	Neg
1	B02	Pos
1	B03	Sample
1	B04	Sample

Lines from the example plate configuration file. Each 384-well plate contains dsRNAs against GFP as a negative control in well B01 and against the mRNA for the antiapoptotic IAP protein as a positive control in well *B*02. ds, double-stranded; GFP, green fluorescent protein; IAP, inhibitor of apoptosis.

**Table 2:** Primary data file

Well coordinate	Luminescence value
A01	887763
A02	958308
A03	1012685
A04	872603
A05	1179875

The first five lines of an example intensity measurement file. In total, it has 384 rows, one for each well in the microtitre plate.

**Table 3:** Plate list file

Filename	Plate	Replicate
FT01-G01.txt	1	1
FT01-G02.txt	1	2
FT02-G01.txt	2	1
FT02-G02.txt	2	2
FT03-G01.txt	3	1

The first five lines of the example plate list file. In total, it has 114 rows, corresponding to 57 plates with two replicates each. The reporter column is omitted because there is only one reporter in this experiment.

**Table 4:** Library annotation file

Plate	Well	HfaID	GeneID
1	A03	HFA00274	CG11371
1	A04	HFA00646	CG31671
1	A05	HFA00307	CG11376
1	A06	HFA00324	CG11723

The first lines of the example library annotation file. It lists the set of dsRNAs in the library (here, identified by an internal Amplicon ID and by the CG identifier of the target gene) together with the specification of the plate and well into which they were arrayed.

**Table 5:** Screen log file

Filename	Well	Flag	Comment
FT06-G01.txt	A01	NA	Contamination
FT06-G02.txt	A01	NA	Contamination
FT06-G01.txt	A02	NA	Contamination

The first lines of the example screen log file. It can be used to flag individual measurements for exclusion from the analysis.

**Table 6:** Category analysis

*n*	*z*_mean_	*P*	GO category	Description
113	2.5	2 × 10^-19^	5840 (CC)	Ribosome
81	1.8	4 × 10^-9^	5829 (CC)	Cytosol
45	2.8	1 × 10^-8^	0502 (CC)	Proteasome complex
284	1.2	3 × 10^-18^	6412 (BP)	Protein biosynthesis
96	0.9	1 × 10^-5^	6397 (BP)	mRNA processing
24	2.2	0.0002	4298 (MF)	Threonine endopeptidase activity
57	0.8	0.0009	8135 (MF)	Translation factor activity, nucleic acid binding

Selected GO categories whose member genes had particularly high *z*-scores. GO, Gene Ontology; *n*, number of genes annotated with that category and targeted by the RNAi library; *P*, *P *value for the null hypothesis that the mean *z*-score of the dsRNAs for this category is the same as that of all dsRNAs; RNAi, RNA interference; *z*_mean_, mean *z*-score.
