# Supplementary material for: Analysis of cell-based RNAi screens
Source: Genome Biol. 2006 Jul 25;7(7):R66. doi: 10.1186/gb-2006-7-7-r66 (PMC1779553; doi:10.1186/gb-2006-7-7-r66)
Supplement: Additional data file 2 — R package in "Windows binary" format. This file archive also contains the example data. [file gb-2006-7-7-r66-S2.zip › cellHTS/doc/cellhts.pdf]

End-to-end analysis of cell-based screens: from raw intensity readings to the annotated hit list

Michael Boutros, Lígia Brás and Wolfgang Huber

August 6, 2006

Contents

1 Introduction 2

2 Reading the intensity data 2

3 The *cellHTS* class and reports 4

4 Annotating the plate results 5
4.1 Format of the plate configuration file 6
4.1.1 Multiple plate configurations 7
4.2 Format of the screen log file 7

5 Normalization and summarization of replicates 7
5.1 Alternative processing strategies 9

6 Annotation 10
6.1 Adding additional annotation from public databases 10
6.1.1 Installation 11
6.1.2 Using biomaRt to annotate the target genes online . . 11

7 Report 14
7.1 Exporting data to a tab-delimited file 16

8 Category analysis 17

9 Comparison with the results previously reported 18

10 Appendix: Data transformation 20

1 Introduction

This is a technical report that demonstrates the use of the *cellHTS* package. It accompanies the paper *Analysis of cell-based RNAi screens* by Michael Boutros, Lígia Brás and Wolfgang Huber. This report explains all the steps necessary to run a complete analysis of a cell-based high-throughput screen (HTS), from raw intensity readings to an annotated hit list.

This text has been produced as a reproducible document [5]. It contains the actual computer instructions for the method it describes, and these in turn produce all results, including the figures and tables that are shown here. The computer instructions are given in the language R, thus, in order to reproduce the computations shown here, you will need an installation of R (version 2.3 or greater) together with a recent version of the package *cellHTS* and of some other add-on packages.

To reproduce the computations shown here, you do not need to type them or copy-paste them from the PDF file; rather, you can take the file *cellhts.Rnw* in the *doc* directory of the package, open it in a text editor, run it using the R command *Sweave*, and modify it to your needs.

First, we load the package.

```
> library("cellHTS")
```

2 Reading the intensity data

We consider a cell-based screen that was conducted in microtiter plate format, where a library of double-stranded RNAs was used to target the corresponding genes in cultured *Drosophila* Kc_{167} cells [2]. Each of the wells in the plates contains either a gene-specific probe, a control, or it can be empty. The experiments were done in duplicate, and the viability of the cells after treatment was recorded by a plate reader measuring luciferase activity, which is indicative of ATP levels. Although this set of example data corresponds to a single-channel screening assay, the *cellHTS* package can also deal with cases where there are readings from more channels, corresponding to different reporters. Usually, the measurements from each replicate and each channel come in individual result files. The set of available result files and the information about them (which plate, which replicate, which channel) is contained in a spreadsheet, which we call the *plate list file*. This file should contain the following columns: *Filename*,*Plate*, and *Replicate*. The last two columns should be numeric, with values ranging from 1 to the

Filename	Plate	Replicate	Channel
RA01D1.TXT	1	1	1
RA01D2.TXT	1	2	1
RA02D1.TXT	2	1	1
RA02D2.TXT	2	2	1
RA03D1.TXT	3	1	1
...	...	...	...

Table 1: Selected lines from the example plate list file `Platelist.txt`.

maximum number of plates or replicates, respectively. The first few lines of an example plate list file are shown in Table 1.

The first step of the analysis is to read the plate list file, to read all the intensity files, and to assemble the data into a single R object that is suitable for subsequent analyses. The main component of that object is one big table with the intensity readings of all plates, channels, and replicates. We demonstrate the R instructions for this step. First we define the path where the input files can be found.

```
> experimentName = "KcViab"
> dataPath = system.file(experimentName, package = "cellHTS")
```

In this example, the input files are in the `KcViab` directory of the *cellHTS* package. To read your own data, modify `dataPath` to point to the directory where they reside. We show the names of 12 files from our example directory:

```
> dataPath

[1] "D:/biocbld/1.9d/tmpdir/Rinst451241720/cellHTS/KcViab"

> rev(dir(dataPath))[1:12]

 [1] "Screenlog.txt"          "Platelist.txt"          "Plateconf.txt"
 [4] "GeneIDs_Dm_HFA_1.1.txt" "FT57-G02.txt"           "FT57-G01.txt"
 [7] "FT56-G02.txt"           "FT56-G01.txt"           "FT55-G02.txt"
[10] "FT55-G01.txt"           "FT54-G02.txt"           "FT54-G01.txt"
```

and read the data into the object `x`

```
> x = readPlateData("Platelist.txt", name = experimentName, path = dataPath)

> x
```

```
cellHTS object of name 'KcViab'
57 plates with 384 wells, 2 replicates, 1 channel. State:
configured normalized     scored  annotated
     FALSE      FALSE      FALSE      FALSE
```

The plate format used in the screen (96-well or 384-well plate design) is automatically determined from the raw intensity files, when calling the *readPlateData* function.

3 The *cellHTS* class and reports

The basic data structure of the package is the class *cellHTS*. In the previous section, we have created the object `x`, which is an instance of this class. All subsequent analyses, such as normalization, gene selection and annotation, will add their results into this object. Thus, the complete analysis project is contained in this object, and a complete dataset can be shared with others and stored for subsequent computational analyses in the form of such an object. In addition, the package offers export functions for generating human-readable reports, which consist of linked HTML pages with tables and plots. The final scored hit list is written as a tab-delimited format suitable for reading by spreadsheet programs.

To create a report, use the function *writeReport*. It will create a directory of the name given by `x$name` in the working directory. Alternatively, the argument `outdir` can be specified to direct the output to another directory.

```
> out = writeReport(x)
```

It can take a while to run this function, since it writes a large number of graphics files. After this function has finished, the index page of the report will be in the file indicated by the variable `out`,

```
> out
```

```
[1] "D:/biocbld/1.9d/Rpacks/cellHTS/inst/doc/KcViab/index.html"
```

and you can view it by directing a web browser to that file.

```
> browseURL(out)
```

Batch	Well	Content
1	A01	geneA
1	A02	geneB
1	A03	sample
1	A04	sample
...	...	...

Table 2: Selected lines from the example plate configuration file `Plate-conf.txt`.

Filename	Well	Flag	Comment
RA03D1.TXT	A05	NA	contaminated
RB03D1.TXT	A05	NA	contaminated
...	...	...	...

Table 3: Selected lines from the example screen log file `Screenlog.txt`.

4 Annotating the plate results

The next step of the analysis is to annotate the measured data with information on controls and to flag invalid measurements. The software expects the information on the controls in a so-called *plate configuration file* (see Section 4.1). This is a tab-delimited file with one row per well.

```
> confFile = file.path(dataPath, "Plateconf.txt")
```

Selected lines of this file are shown in Table 2. Individual measurements can be flagged as invalid in the so-called *screen log file* (see Section 4.2).

```
> logFile = file.path(dataPath, "Screenlog.txt")
```

The first 5 lines of this file are shown in Table 3. The *screen description* file contains a general description of the screen, its goal, the conditions under which it was performed, references, and any other information that is pertinent to the biological interpretation of the experiments.

```
> descripFile = file.path(dataPath, "Description.txt")
```

We now apply this information to the data object `x`.

```
> x = configure(x, confFile, logFile, descripFile)
```

Note that the function *configure*[1] takes `x`, the result from Section 2, as an argument, and we then overwrite `x` with the result of this function. If no screen log file is available for the experiment, the argument `logFile` of the function *configure* should be ommitted.

4.1 Format of the plate configuration file

The software expects this to be a rectangular table in a tabulator delimited text file, with mandatory columns *Batch*, *Well*, *Content*. The *Batch* column allows to have different plate configurations (see Section 4.1.1). The *Well* column contains the name of each well of the plate, in letter-number format (in this case, `A01` to `P24`). As the name suggests, the *Content* column provides the content of each well in the plate (here referred to as the *well annotation*). Mainly, this annotation falls into four categories: empty wells, wells containing genes of interest, control wells, and wells containing other things that do not fit in the previous categories. The first two types of wells should be indicated in the *Content* column of the plate configuration file by *empty* and *sample*, respectively, while the last type of wells should be indicated by *other*. The designation for the control wells in the *Content* conlumn is more flexible. By default, the software expects them to be indicated by *pos* (for positive controls), or *neg* (for negative controls). However, other names are allowed, given that they are specified by the user whenever necessary (for example, when calling the *writeReport* function). This versatility for the control wells' annotation is justified by the fact that,sometimes, multiple positive and/or negative controls can be employed in a given screen, making it useful to give different names to the distinct controls in the *Content* column. Moreover, this versatility is also required in multi-channel screens for which we frequently have reporter-specific controls. Note that the well annotations mentioned above are used by the software in the normalization, quality control, and gene selection calculations. Data from wells that are annotated as *empty* are ignored, i. e. they are set to `NA`. Here we look at the frequency of each well annotation in the example data:

```
> table(x$plateConf$Content)

   neg  other    pos sample 
     1      2      1    380 
```

[1]More precisely, *configure* is a method for the S3 class *cellHTS*.

4.1.1 Multiple plate configurations

Although it is good practice to use the same plate configuration for the whole experiment, sometimes this does not work out, and there are different parts of the experiment with different plate configurations. It is possible to specify multiple plate configurations simply by appending them to each other in the plate configuration file, and marking them with different numbers in the column *Batch*.

Note that replicated experiments per plate have to use the same plate configuration.

4.2 Format of the screen log file

The screen log file is a tabulator delimited file with mandatory columns *Filename*, *Well*, *Flag*. In addition, it can contain arbitrary optional columns. Each row corresponds to one flagged measurement, identified by the filename and the well identifier. The type of flag is specified in the column *Flag*. Most commonly, this will have the value "NA", indicating that the measurement should be discarded and regarded as missing.

5 Normalization and summarization of replicates

The function *normalizePlates* can be called to adjust for plate effects. Its parameter `normalizationMethod` allows to choose between different types of normalization. For example, if it is set to `"median"`, the function *normalizePlates* adjusts for plate effects by dividing each value in each plate by the median of values in the plate:

$$x'_{ki} = \frac{x_{ki}}{M_i} \qquad \forall k, i \tag{1}$$

$$M_i = \operatorname*{median}_{m \in \text{samples}} x_{mi} \tag{2}$$

where x_{ki} is the raw intensity for the k-th well in the i-th replicate file, and x'_{ki} is the corresponding normalized intensity. The median is calculated across the wells annotated as *sample* in the i-th result file. This is achieved by calling

```
> x = normalizePlates(x, normalizationMethod = "median")
```

after which the normalized intensities are stored in the slot `x$xnorm`. This is an array of the same size as `x$xraw`.

We can now summarize the replicates, calculating a single score for each gene. One option would be to take the root mean square of the values from the replicates:

$$z_{ki} = \pm \frac{x'_{ki} - \hat{\mu}}{\hat{\sigma}} \tag{3}$$

$$z_k = \sqrt{\frac{1}{n_{\text{rep}_k}} \sum_{r=1}^{n_{\text{rep}_k}} z_{kr}^2}. \tag{4}$$

Before summarizing the replicate, we standardize the values for each replicate experiment using Equation (3). Here $\hat{\mu}$ and $\hat{\sigma}$ are estimators of location and scale of the distribution of x'_{ki} taken across all plates and wells of a given replicate experiment. We use robust estimators, namely, median and median absolute deviation (MAD). Moreover, we only consider the wells containing "sample" for estimating $\hat{\mu}$ and $\hat{\sigma}$. As the values x'_{ki} were obtained using plate median normalization (1), it holds that $\hat{\mu} = 1$. The symbol $\pm$ indicates that we allow for either plus or minus sign in equation (3); the minus sign can be useful in the application to an inhibitor assay, where an effect results in a decrease of the signal and we may want to see this represented by a large z-score. Then, in Equation (4), the summary is taken over all the n_{rep_k} replicates of probe k.

Depending on the intended stringency of the analysis, other plausible choices of summary function between replicates are the minimum, the maximum, and the mean. In the first case, the analysis would be particularly conservative: all replicate values have to be high in order for z_k to be high. In order to compare our results with those obtained in the paper of Boutros *et al.* [2], we choose to consider the mean as a summary:

```
> x = summarizeReplicates(x, zscore = "-", summary = "mean")
```

The resulting single z-score value per probe will be stored in the slot `x$score`. Boxplots of the z-scores for the different types of probes are shown in Figure 1.

```
> ylim = quantile(x$score, c(0.001, 0.999), na.rm = TRUE)
> boxplot(x$score ~ x$wellAnno, col = "lightblue", outline = FALSE,
+     ylim = ylim)
```

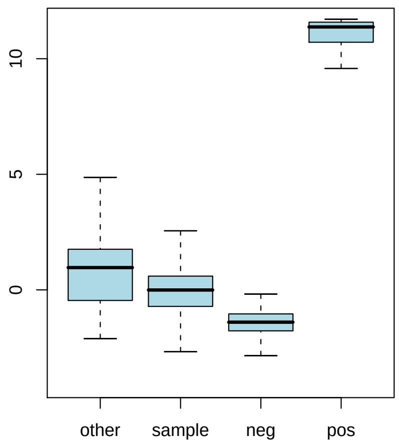

Figure 1: Boxplots of z-scores for the different types of probes.

5.1 Alternative processing strategies

The HTML quality report will consider the values in the slot `x$xnorm` for the calculation of its quality metrics. In the example above, `x$xnorm` contains the data after plate median normalization, but before calculation of the z-scores and the multiplication by -1. The package *cellHTS* allows some flexibility with respect to these steps. We can already calculate the z-scores and multiply by -1 in the function *normalizePlates*, and then do the summarization between replicates, by calling the function *summarizeReplicates* without the argument `zscore`.

```
> xalt = normalizePlates(x, normalizationMethod = "median", zscore = "-")
> xalt = summarizeReplicates(xalt, summary = "mean")
```

It is easy to define alternative normalization methods, for example, to adjust for additional experimental biases besides the plate effect. You might want to start by taking the source code of *normalizePlates* as a template.

**Plate**	**Well**	**HFAID**	**GeneID**
1	A03	HFA00274	CG11371
1	A04	HFA00646	CG31671
1	A05	HFA00307	CG11376
1	A06	HFA00324	CG11723
...	...	...	...

Table 4: Selected lines from the example gene ID file `GeneIDs_Dm_HFA_1.1.txt`.

6 Annotation

Up to now, the assayed genes have been identified solely by the identifiers of the plate and the well that contains the probe for them. The *annotation file* contains additional annotation, such as the probe sequence, references to the probe sequence in public databases, the gene name, gene ontology annotation, and so forth. Mandatory columns of the annotation file are *Plate*, *Well*, and *GeneID*, and it has one row for each well. The content of the *GeneID* column will be species- or project-specific. The first 5 lines of the example file are shown in Table 4, where we have associated each probe with CG-identifiers for the genes of *Drosophila melanogaster*.

```
> geneIDFile = file.path(dataPath, "GeneIDs_Dm_HFA_1.1.txt")
> x = annotate(x, geneIDFile)
```

6.1 Adding additional annotation from public databases

For the analysis of the RNAi screening results, we usually want to consider gene annotation information such as Gene Ontology, chromosomal location, gene function summaries, homology. The package *biomaRt* can be used to obtain such annotation from public databases [3]. However, there are also numerous alternative methods to annotate a list of gene identifiers with public annotation – pick your favourite one.

This section demonstrates how to do it with the package *biomaRt*. It is optional, you can move on to Section 7 if you do not have the *biomaRt* package or do not want to use it. If you do skip this section, then for the purpose of this vignette, please load a cached version of the gene annotation:

```
> data("bdgpbiomart")
> x$geneAnno = bdgpbiomart
```

6.1.1 Installation

The installation of the *biomaRt* package can be a little bit tricky, since it relies on the two packages *RCurl* and *XML*, which in turn rely on the presence of the system libraries *libcurl* and *libxml2* on your computer. If you are installing the precompiled R packages (for example, this is what most people do on Windows), then you need to make sure that the system libraries on your computer are version-compatible with those on the computer where the R packages were compiled, and that they are found. If you are installing the R packages from source, then you need to make sure that the library header files are available and that the headers as well as the actual library is found by the compiler and linker. Please refer to the *Writing R Extensions* manual and to the FAQ lists on www.r-project.org.

6.1.2 Using biomaRt to annotate the target genes online

In the remainder of this section, we will demonstrate how to obtain the dataframe `bdgpbiomart` by querying the online webservice *BioMart* and through it the Ensembl genome annotation database [1].

```
> library("biomaRt")
```

By default, the *biomaRt* package will query the webservice at http://www.ebi.ac.uk/biomart/martservice. Let us check which BioMart databases it covers:

```
> listMarts()

$biomart
[1] "dicty"    "ensembl"  "snp"      "vega"     "uniprot"  "msd"      "wormbase"

$version
[1] "DICTYBASE (NORTHWESTERN)" "ENSEMBL 38 (SANGER)"
[3] "SNP 38 (SANGER)"          "VEGA 38 (SANGER)"
[5] "UNIPROT 4-5 (EBI)"        "MSD 4 (EBI)"
[7] "WORMBASE CURRENT (CSHL)"

$host
[1] "www.dictybase.org" "www.biomart.org"   "www.biomart.org"
[4] "www.biomart.org"   "www.biomart.org"   "www.biomart.org"
[7] "www.biomart.org"
```

```
$path
[1] ""                       "/biomart/martservice" "/biomart/martservice"
[4] "/biomart/martservice" "/biomart/martservice" "/biomart/martservice"
[7] "/biomart/martservice"
```

In this example, we use the Ensembl database [1], from which we select the *D. melanogaster* dataset.

```
> mart = useMart("ensembl")

> listDatasets(mart = mart)

                     dataset    version
1    rnorvegicus_gene_ensembl    RGSC3.4
2    scerevisiae_gene_ensembl       SGD1
3       celegans_gene_ensembl     CEL150
4  cintestinalis_gene_ensembl       JGI2
5   ptroglodytes_gene_ensembl    CHIMP1A
6      frubripes_gene_ensembl      FUGU4
7       agambiae_gene_ensembl     AgamP3
8       hsapiens_gene_ensembl     NCBI36
9        ggallus_gene_ensembl    WASHUC1
10   xtropicalis_gene_ensembl     JGI4.1
11        drerio_gene_ensembl     ZFISH5
12 tnigroviridis_gene_ensembl TETRAODON7
13      mmulatta_gene_ensembl   MMUL_0_1
14    mdomestica_gene_ensembl    BROADO2
15    amellifera_gene_ensembl    AMEL2.0
16 dmelanogaster_gene_ensembl    BDGP4.2
17     mmusculus_gene_ensembl    NCBIM35
18       btaurus_gene_ensembl   Btau_2.0
19   cfamiliaris_gene_ensembl    BROADD1

> mart = useDataset("dmelanogaster_gene_ensembl", mart)
```

We can query the available gene attributes and filters for the selected dataset using the following functions.

```
> attrs = listAttributes(mart)
> filts = listFilters(mart)
```

In the BioMart system [8], a *filter* is a property that can be used to select a gene or a set of genes (like the "where" clause in an SQL query), and an *attribute* is a property that can be queried (like the "select" clause in an SQL query). We use the *getBM* function of the package *biomaRt* to obtain the gene annotation from Ensembl.

```
> myGetBM = function(att) getBM(attributes = c("ensembl_gene_id",
+     att), filter = "gene_stable_id", values = unique(x$geneAnno$GeneID),
+     mart = mart)
```

For performance reasons, we split up our query in three subqueries, which corresponds to different areas in the BioMart schema, and then assemble the results together in R. Alternatively, it would also be possible to submit a single query for all of the attributes, but then the result table will be enormous due to the 1:many mapping especially from gene ID to GO categories [6].

```
> bm1 = myGetBM(c("chromosome_name", "start_position", "end_position",
+     "description"))
> bm2 = myGetBM(c("flybase_name"))
> bm3 = myGetBM(c("go", "go_description"))
```

There are only a few CG-identifiers for which we were not able to obtain chromosomal locations:

```
> unique(setdiff(x$geneAnno$GeneID, bm1$ensembl_gene_id))
```

```
 [1] NA        "CG7245"  "CG6735"  "CG31314" "CG15509" "CG15388" "CG15389"
 [8] "CG11169" "CG18648" "CG13459" "CG15507"
```

Below, we add the results to the dataframe `x$geneAnno`. Since the tables `bm1`, `bm2`, and `bm3` contain zero, one or several rows for each gene ID, but in `x$geneAnno` we want exactly one row per gene ID, the function *oneRowPerId* does the somewhat tedious task of reformatting the tables: multiple entries are collapsed into a single comma-separated string, and empty rows are inserted where necessary.

```
> id = x$geneAnno$GeneID
> bmAll = cbind(oneRowPerId(bm1, id), oneRowPerId(bm2, id), oneRowPerId(bm3,
+     id))
> bdgpbiomart = cbind(x$geneAnno, bmAll)
> x$geneAnno = bdgpbiomart
```

7 Report

We have now completed the analysis tasks: the dataset has been read, configured, normalized, scored, and annotated:

```
> x

cellHTS object of name 'KcViab'
57 plates with 384 wells, 2 replicates, 1 channel. State:
configured normalized     scored  annotated
      TRUE       TRUE       TRUE       TRUE
```

We can now save the data set to a file.

```
> save(x, file = paste(experimentName, ".rda", sep = ""), compress = TRUE)
```

The dataset can be loaded again for subsequent analysis, or passed on to others. To produce a comprehensive report, we can call the function *writeReport* again,

```
> out = writeReport(x, force = TRUE, plotPlateArgs = list(xrange = c(0.5,
+     1.5)), imageScreenArgs = list(zrange = c(-2, 6.5), ar = 1))
```

and use a web browser to view the resulting report

```
> browseURL(out)
```

Now, the report contains a quality report for each plate, and also for the whole screening assays. The experiment-wide report presents the Z'-factor determined for each experiment (replicate) using the positive and negative controls [9], the boxplots with raw and normalized intensities for the different plates, and the screen-wide plot with the z-scores in every well position of each plate. The latter image plot can also be produced separately using the function *imageScreen* given in the *cellHTS* package. This might be useful if we want to select the best display for our data, namely, the aspect ratio for the plot and/or the range of z-score values to be mapped into the color scale. These can be passed to the function's arguments `ar` and `zrange`, respectively. For example,

```
> imageScreen(x, ar = 1, zrange = c(-3, 4))
```

It should be noted that the per-plate and per-experiment quality reports are constructed based on the content of `x$xnorm`, if it is present in the `x`

object. Otherwise, it uses the content given in the slot `x$xraw`. In the case of dual-channel experiments, the `x$xnorm` slot could also contain the ratio between the intensities in two different channels, etc. The main point that we want to highlight is that `x$xnorm` should contain the data that we want to visualize in the HTML quality reports. On the other hand, `x$score` should always contain the final list of scored probes (one value per probe).

The quality report produced by *writeReport* function has also a link to a file called *topTable.txt* that contains the list of scored probes ordered by decreasing z-score values. This file has one row for each well and plate, and for the present example data set, it has the following columns:

- `plate`;
- `position` gives the position of the well in the plate (runs from 1 to the total number of wells in the plate);
- `score` corresponds to the score calculated for the probe (content of `x$score`);
- `wellAnno` corresponds to the well annotation (as given by the plate configuration file;
- `normalized_r1_ch1` and `normalized_r2_ch1` give the normalized intensities for replicate 1 and replicate 2, respectively ('ch' refers to channel). This corresponds to the content of `x$xnorm`;
- `finalWellAnno_r1_ch1` and `finalWellAnno_r2_ch1` give the final well annotation for replicate 1 and 2, respectively, which combines the information given in the plate configuration file and the screen log file, so that the wells that were flagged in the latter file appear with the annotation *flagged*. This information corresponds to the content of `x$finalWellAnno`;
- `raw_r1_ch1` and `raw_r2_ch1` contain the raw intensities for replicate 1 and replicate 2, respectively (content of `x$xraw`);
- `median_ch1` corresponds to the median of raw measurements across replicates;
- `diff_ch1` gives the difference between replicated raw measurements (only given if the number of replicates is equal to two);
- `average_ch1` corresponds to the average between replicated raw intensities (only given if the number of replicates is higher than two);

- `raw/PlateMedian_r1_ch1` and `raw/PlateMedian_r2_ch1` give the ratio between each raw measurement and the median intensity in each plate for replicate 1 and replicate 2, respectively. The plate median is determined for the raw intensities, using exclusively the wells annotated as "sample".

Additionally, if `x` has been annotated (as in the present case), it also contains the data given in the original gene anotation file that was stored in `x$geneAnno`.

7.1 Exporting data to a tab-delimited file

The *cellHTS* package contains a function called *writeTab* to save `x$xraw` and, if available, `x$xnorm` data from a *cellHTS* object to a tab-delimited file to a file. The rows of the file are sorted by plate and well, and there is one row for each plate and well. Its columns correspond to the content of `x$geneAnno` (that is, the gene annotation information), together with the raw measurements, and if available, the normalized intensities for each replicate and channel. The name for the columns containing the raw intensities starts with "R" and is followed by the replicate identifier "r", and by the channel identifier "c". For example, `Rr2c1` refers to the raw data for replicate 2 in channel 1. For the normalized data, the column names start with "N" instead of "R".

```
> writeTab(x, file = "Data.txt")
```

Since you might be interestered in saving other values to a tab delimited file, below we demonstrate how you can create a matrix with the ratio between each raw measurement and the plate median, together with the gene and well annotation, and export it to a tab-delimited file using the function *write.tabdel* [2] also provided in the *cellHTS* package.

```
> y = array(as.numeric(NA), dim = dim(x$xraw))
> nrWell = dim(x$xraw)[1]
> for (p in 1:(dim(x$xraw)[2])) {
+     samples = (x$wellAnno[(1:nrWell) + nrWell * (p - 1)] == "sample")
+     y[, p, , ] = apply(x$xraw[, p, , , drop = FALSE], 3:4, function(w) w/median(w[s
+         na.rm = TRUE))
+ }
```

[2]This function is a wrapper of the function *write.table*, whereby you just need to specify the name of the data object and the file

```
> y = signif(y, 4)
> out = matrix(y, nrow = prod(dim(y)[1:2]), ncol = dim(y)[3:4])
> out = cbind(x$geneAnno, x$wellAnno, out)
> colnames(out) = c(names(x$geneAnno), "wellAnno", sprintf("Well/Median_r%d_ch%d",
+     rep(1:dim(y)[3], dim(y)[4]), rep(1:dim(y)[4], each = dim(y)[3])))
> write.tabdel(out, file = "WellMedianRatio.txt")
```

At this point we are finished with the basic analysis of the screen. As one example for how one could continue to further mine the screen results for biologically relevant patterns, we demonstrate an application of category analysis.

8 Category analysis

We would like to see whether there are Gene Ontology categories [6] overrepresented among the probes with a high score. For this we use the category analysis from Robert Gentleman's *Category* package [4]. Similar analyses could be done for other categorizations, for example chromosome location, pathway membership, or categorical phenotypes from other studies.

```
> library("Category")
```

Now we can create the category matrix. Conceptually, this a matrix with one column for each probe and one row for each category. The matrix element `[i,j]` is `1` if probe `j` belongs to the `j`-th category, and `0` if not. In practice, this matrix would be rather large (and perhaps exhaust the memory of your computer), hence we use a type of sparse matrix representation, implemented by the *graph* object `categs`. This bipartite graph's nodes correspond to the rows and columns of the matrix and there is an edge if the matrix element is non-zero.

One distraction is the GO term GO:0000067, which is annotated to some of the genes, but is obsolete.

```
> names(x$score) = x$geneAnno$GeneID
> sel = !is.na(x$score) & (!is.na(x$geneAnno$go))
> goids = strsplit(x$geneAnno$go[sel], ", ")
> goids = lapply(goids, function(x) x[x != "GO:0000067"])
> genes = rep(x$geneAnno$GeneID[sel], listLen(goids))
> cache(categs <- cateGOry(genes, unlist(goids, use.names = FALSE)))
```

We will select only those categories that contain at least 3 and no more than 1000 genes.

```
> remGO = which(regexpr("^GO:", nodes(categs)) > 0)
> nrMem = listLen(edges(categs)[remGO])
> remGO = remGO[nrMem > 1000 | nrMem < 3]
> categs = subGraph(nodes(categs)[-remGO], categs)
```

As the statistic for the category analysis we use the z-score. After selecting the subset of genes that actually have GO annotation,

```
> stats = x$score[sel & (names(x$score) %in% nodes(categs))]
```

we are ready to call the category summary functions:

```
> acMean = applyByCategory(stats, categs)
> acTtest = applyByCategory(stats, categs, FUN = function(v) t.test(v,
+     stats)$p.value)
> acNum = applyByCategory(stats, categs, FUN = length)
> isEnriched = (acTtest <= 0.001) & (acMean > 0.5)
```

A volcano plot of the $-\log_{10}$ of the p-value `acTtest` versus the per category mean z-score `acMean` is shown in Figure 2. For a given category, the p-value is calculated from the t-test against the null hypothesis that there is no difference between the mean z-score of all probes and the mean z-score of the probes in that category. To select the enriched categories (`isEnriched`), we considered a significance level of 0.1% for the t-test, and a per category mean z-score greater than 0.5. This led to the 34 categories marked in red in Figure 2 are listed in Table 5.

9 Comparison with the results previously reported

In this section we compare the current results obtained using *cellHTS* package, with the ones previously reported in Boutros *et al.* [2]. The file "Analysis2003.txt" in the same directory as the input data files, i. e. in `KcViab` directory of the *cellHTS* package. First, We will load this file:

```
> data2003 = read.table(file.path(dataPath, "Analysis2003.txt"),
+     header = TRUE, as.is = TRUE, sep = "\t")
```

The file contains the columns `Plate, Position, Score, Well, HFAID, GeneID`. The scored values in the `Scores` column will be compared with the ones obtained in our analysis. For that, I will start by adding to `data2003`, a column with the corresponding z-score values calculated using the *cellHTS* package.

n	$z_{\mathbf{mean}}$	p	**GOID**	**Ontology**	**description**
113	2.5	1.5e-19	GO:0005840	CC	ribosome
175	1.8	4.1e-19	GO:0030529	CC	ribonucleoprotein complex
694	0.75	6e-19	GO:0043234	CC	protein complex
487	0.78	8e-15	GO:0043228	CC	non-membrane-bound organelle
487	0.78	8e-15	GO:0043232	CC	intracellular non-membrane-bound organelle
81	1.8	3.8e-09	GO:0005829	CC	cytosol
45	2.8	9.9e-09	GO:0000502	CC	proteasome complex (sensu Eukaryota)
19	4	2.8e-06	GO:0005838	CC	proteasome regulatory particle (sensu Eukaryota)
24	2.2	0.00023	GO:0005839	CC	proteasome core complex (sensu Eukaryota)
13	2.7	0.00087	GO:0015934	CC	large ribosomal subunit
284	1.2	3.3e-18	GO:0006412	BP	protein biosynthesis
325	1	1e-16	GO:0009059	BP	macromolecule biosynthesis
562	0.71	1.7e-15	GO:0044249	BP	cellular biosynthesis
626	0.59	1.6e-12	GO:0009058	BP	biosynthesis
66	1	9.8e-06	GO:0000375	BP	RNA splicing, via transesterification reactions
66	1	9.8e-06	GO:0000377	BP	RNA splicing, via transesterification reactions with bulged adenosine as nucleophile
66	1	9.8e-06	GO:0000398	BP	nuclear mRNA splicing, via spliceosome
96	0.87	1.2e-05	GO:0006397	BP	mRNA processing
101	0.83	1.2e-05	GO:0016071	BP	mRNA metabolism
70	0.98	1.5e-05	GO:0008380	BP	RNA splicing
167	0.56	2.7e-05	GO:0006396	BP	RNA processing
45	1.1	0.00042	GO:0048024	BP	regulation of nuclear mRNA splicing, via spliceosome
45	1.1	0.00042	GO:0050684	BP	regulation of mRNA processing
46	1	0.00045	GO:0051252	BP	regulation of RNA metabolism
43	1.1	0.00053	GO:0000380	BP	alternative nuclear mRNA splicing, via spliceosome
43	1.1	0.00053	GO:0000381	BP	regulation of alternative nuclear mRNA splicing, via spliceosome
4	1	7e-04	GO:0008335	BP	ovarian ring canal stabilization
114	2.5	1.4e-19	GO:0003735	MF	structural constituent of ribosome
280	0.98	1.4e-12	GO:0005198	MF	structural molecule activity
211	0.57	9.7e-06	GO:0003723	MF	RNA binding
375	0.5	5.7e-05	GO:0030528	MF	transcription regulator activity
24	2.2	0.00023	GO:0004298	MF	threonine endopeptidase activity
57	0.82	0.00089	GO:0008135	MF	translation factor activity, nucleic acid binding
243	0.52	0.00098	GO:0003700	MF	transcription factor activity

Table 5: Top 34 Gene Ontology categories with respect to z-score.

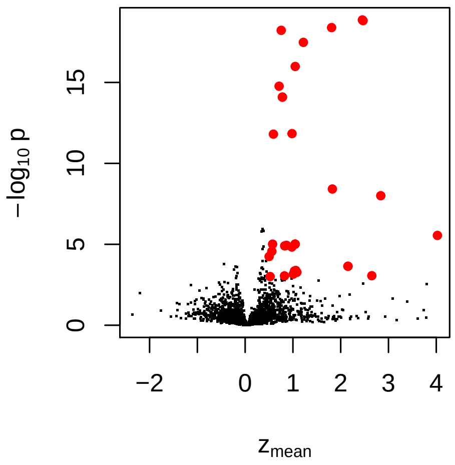

Figure 2: Volcano plot of the t-test p-values and the mean z-values of the category analysis for Gene Ontology categories. The top categories are shown in red.

```
> i = data2003$Position + 384 * (data2003$Plate - 1)
> data2003$ourScore = x$score[i]
```

Figure 3 shows the scatterplot between Boutros *et al.*'s scores and our scores in each of the 384-well plates. The results between the two analyses are very similar, except for two minor details: use of robust estimators of location and spread (median and MAD instead of mean and standard deviation), and estimation of MAD over the whole experiment instead of plate-by-plate. In fact, Figure 3 evidenciates how the scored values exactly agree up to an offset (mean versus median) and scale (standard deviation versus MAD).

10 Appendix: Data transformation

An obvious question is whether to do the statistical analyses on the original intensity scale or on a transformed scale such as the logarithmic one. Many statistical analysis methods, as well as visualizations work better if (to sufficient approximation)

- replicate values are normally distributed,
- the data are evenly distributed along their dynamic range,

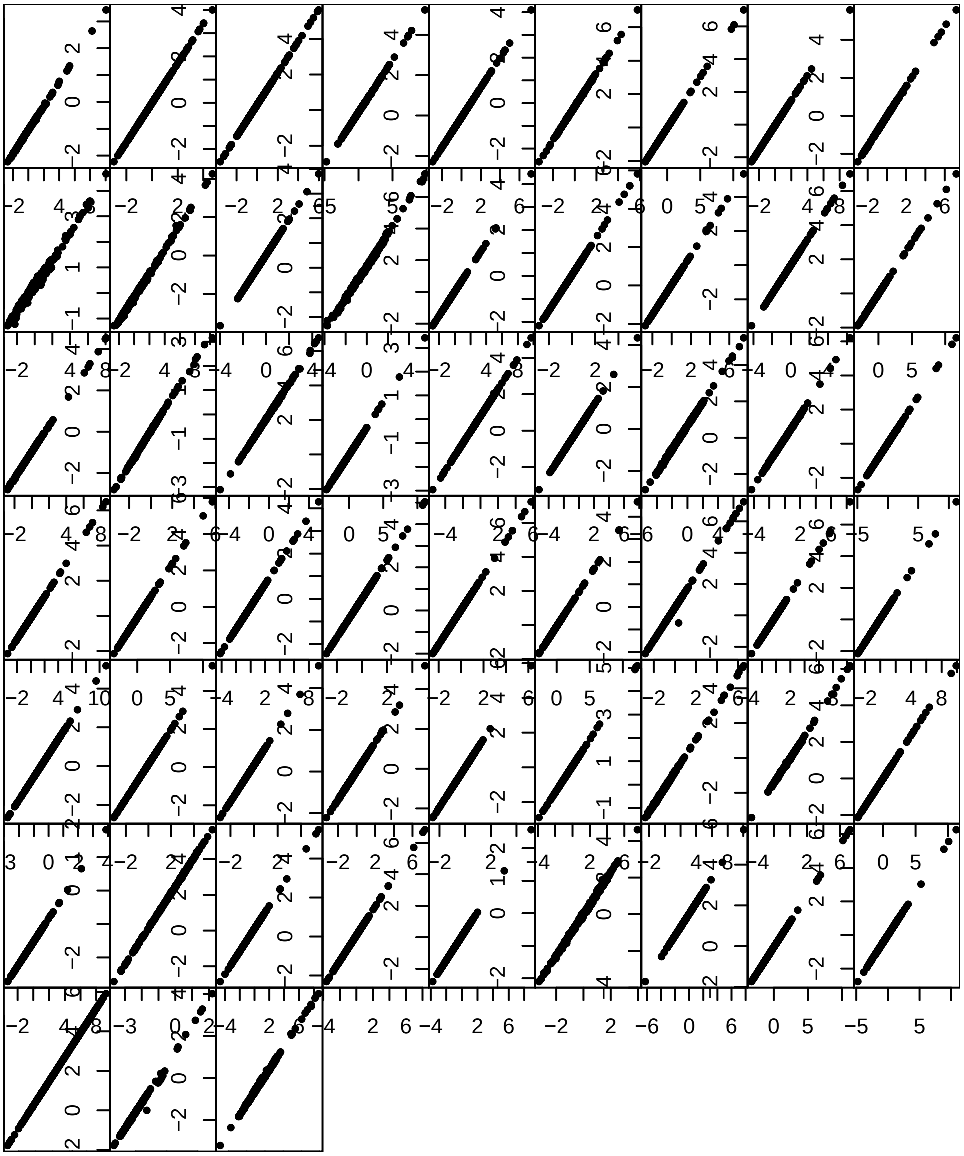

Figure 3: Scored values obtained in the paper of Boutros *et al.* against the scored values calculated herein. Each panel corresponds to one 384-well plate. Axis labels are not pretty - they overlap with neighboring panels due to space constraints.

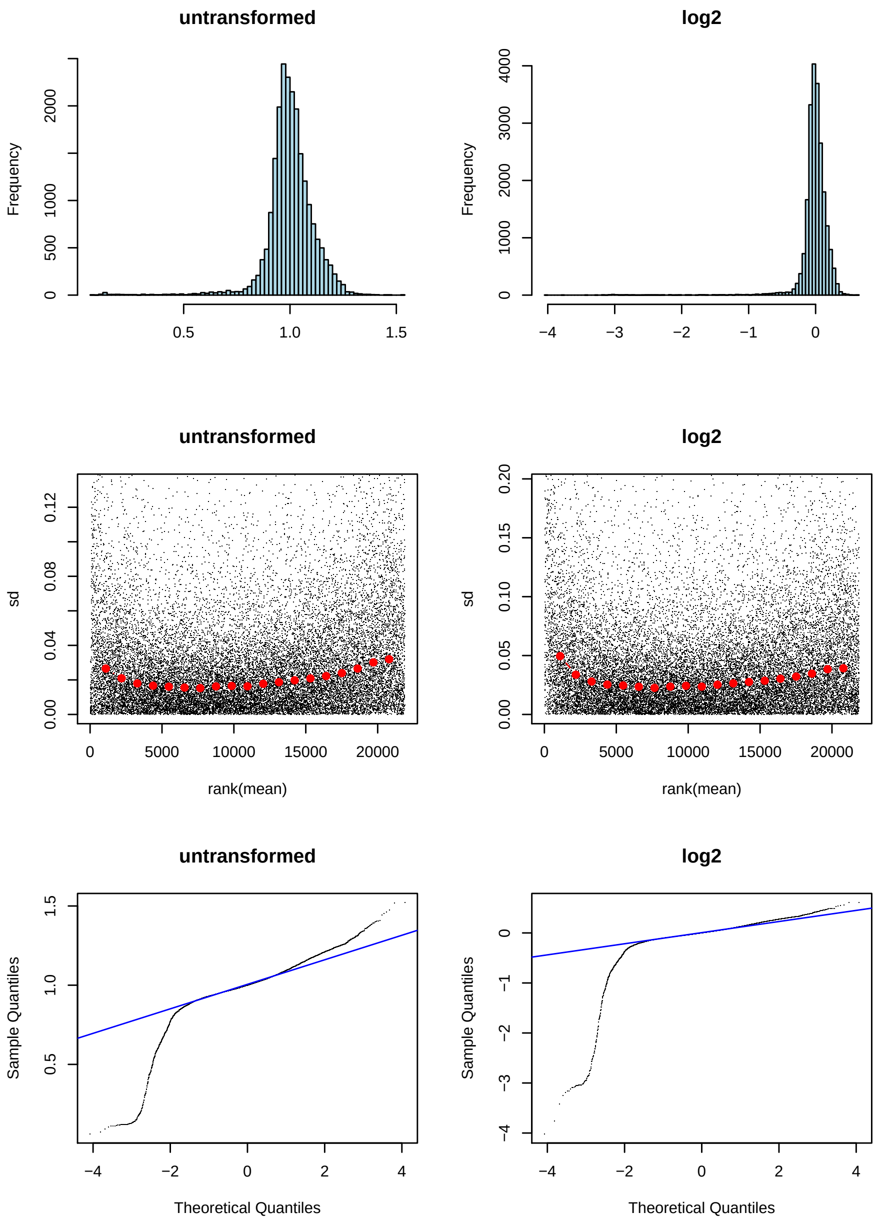

Figure 4: Comparison between untransformed (left) and logarithmically (base 2) transformed (right), normalized data. Upper: histogram of intensity values of replicate 1. Middle: scatterplots of standard deviation versus mean of the two replicates. Bottom: Normal quantile-quantile plots.

- the variance is homogeneous along the dynamic range [7].

Figure 4 compares these properties for untransformed and log-transformed normalized data, showing that the difference is small. Intuitively, this can be explained by the fact that for small x,

$$\log(1 + x) \approx x$$

and that indeed the range of the untransformed data is mostly not far from 1. Hence, for the data examined here, the choice between original scale and logarithmic scale is one of taste, rather than necessity.

```
> library("vsn")
> par(mfcol = c(3, 2))
> myPlots = function(z, ...) {
+     hist(z[, 1], 100, col = "lightblue", xlab = "", ...)
+     meanSdPlot(z, ylim = c(0, quantile(abs(z[, 2] - z[, 1]),
+         0.95, na.rm = TRUE)), ...)
+     qqnorm(z[, 1], pch = ".", ...)
+     qqline(z[, 1], col = "blue")
+ }
> dv = matrix(x$xnorm, nrow = prod(dim(x$xnorm)[1:2]), ncol = dim(x$xnorm)[3])
> myPlots(dv, main = "untransformed")
> xlog = normalizePlates(x, normalizationMethod = "median", transform = log2)
> dvlog = matrix(xlog$xnorm, nrow = prod(dim(xlog$xnorm)[1:2]),
+     ncol = dim(xlog$xnorm)[3])
> myPlots(dvlog, main = "log2")
```

References

[1] E Birney, D Andrews, M Caccamo, Y Chen, L Clarke, G Coates, T Cox, F Cunningham, V Curwen, T Cutts, T Down, R Durbin, X M Fernandez-Suarez, P Flicek, S Graf, M Hammond, J Herrero, K Howe, V Iyer, K Jekosch, A Kahari, A Kasprzyk, D Keefe, F Kokocinski, E Kulesha, D London, I Longden, C Melsopp, P Meidl, B Overduin, A Parker, G Proctor, A Prlic, M Rae, D Rios, S Redmond, M Schuster, I Sealy, S Searle, J Severin, G Slater, D Smedley, J Smith, A Stabenau, J Stalker, S Trevanion, A Ureta-Vidal, J Vogel, S White, C Woodwark, and T J P Hubbard. Ensembl 2006. *Nucleic Acids Res*, 34(Database issue):556–561, Jan 2006. 11, 12

[2] Michael Boutros, Amy A Kiger, Susan Armknecht, Kim Kerr, Marc Hild, Britta Koch, Stefan A Haas, Heidelberg Fly Array Consortium, Renato Paro, and Norbert Perrimon. Genome-wide RNAi analysis of growth and viability in Drosophila cells. *Science*, 303(5659):832–835, Feb 2004. 2, 8, 18

[3] Steffen Durinck, Yves Moreau, Arek Kasprzyk, Sean Davis, Bart De Moor, Alvis Brazma, and Wolfgang Huber. BioMart and Bioconductor: a powerful link between biological databases and microarray data analysis. *Bioinformatics*, 21(16):3439–3440, Aug 2005. 10

[4] R. Gentleman. *Category: Category Analysis*, 2006. R package version 1.3.3. 17

[5] Robert Gentleman. Reproducible research: A bioinformatics case study. *Statistical Applications in Genetics and Molecular Biology*, 3, 2004. 2

[6] M A Harris, J Clark, A Ireland, J Lomax, M Ashburner, R Foulger, K Eilbeck, S Lewis, B Marshall, C Mungall, J Richter, G M Rubin, J A Blake, C Bult, M Dolan, H Drabkin, J T Eppig, D P Hill, L Ni, M Ringwald, R Balakrishnan, J M Cherry, K R Christie, M C Costanzo, S S Dwight, S Engel, D G Fisk, J E Hirschman, E L Hong, R S Nash, A Sethuraman, C L Theesfeld, D Botstein, K Dolinski, B Feierbach, T Berardini, S Mundodi, S Y Rhee, R Apweiler, D Barrell, E Camon, E Dimmer, V Lee, R Chisholm, P Gaudet, W Kibbe, R Kishore, E M Schwarz, P Sternberg, M Gwinn, L Hannick, J Wortman, M Berriman, V Wood, N de la Cruz, P Tonellato, P Jaiswal, T Seigfried, and R White. The Gene Ontology (GO) database and informatics resource. *Nucleic Acids Res*, 32(Database issue):258–261, Jan 2004. 13, 17

[7] Wolfgang Huber, Anja von Heydebreck, Holger Sültmann, Annemarie Poustka, and Martin Vingron. Variance stabilization applied to microarray data calibration and to the quantification of differential expression. *Bioinformatics*, 18 Suppl. 1:S96–S104, 2002. 23

[8] Arek Kasprzyk, Damian Keefe, Damian Smedley, Darin London, William Spooner, Craig Melsopp, Martin Hammond, Philippe Rocca-Serra, Tony Cox, and Ewan Birney. EnsMart: a generic system for fast and flexible access to biological data. *Genome Res*, 14(1):160–169, Jan 2004. 13

[9] JH Zhang, TD Chung, and KR Oldenburg. A Simple Statistical Parameter for Use in Evaluation and Validation of High Throughput Screening Assays. *J Biomol Screen*, 4(2):67–73, 1999. 14
