# Supplementary material for: Analysis of cell-based RNAi screens
Source: Genome Biol. 2006 Jul 25;7(7):R66. doi: 10.1186/gb-2006-7-7-r66 (PMC1779553; doi:10.1186/gb-2006-7-7-r66)
Supplement: Additional data file 2 — R package in "Windows binary" format. This file archive also contains the example data. [file gb-2006-7-7-r66-S2.zip › cellHTS/doc/index.html]

R: cellHTS vignettes

## Vignettes of package cellHTS

cellhts.pdf:: End-to-end analysis of cell-based screens twoChannels.pdf:: Analysis of multi-channel cell-based screens
