# Supplementary material for: Analysis of cell-based RNAi screens
Source: Genome Biol. 2006 Jul 25;7(7):R66. doi: 10.1186/gb-2006-7-7-r66 (PMC1779553; doi:10.1186/gb-2006-7-7-r66)
Supplement: Additional data file 2 — R package in "Windows binary" format. This file archive also contains the example data. [file gb-2006-7-7-r66-S2.zip › cellHTS/doc/twoChannels.pdf]

Analysis of multi-channel cell-based screens

Lígia Brás, Michael Boutros and Wolfgang Huber

August 6, 2006

Contents

1 Introduction **1**

2 Assembling the data **2**
2.1 Reading the raw intensity files 2
2.2 Annotating the plate results 3

3 Data preprocessing and summarization of replicates **4**

1 Introduction

This techical report is a supplement of the main vignette *End-to-end analysis of cell-based screens: from raw intensity readings to the annotated hit list* that is given as part of the *cellHTS* package. It accompanies the paper *Analysis of cell-based RNAi screens* by Michael Boutros, Lígia Brás and Wolfgang Huber.

The report demonstrates how the *cellHTS* package can be applied to the documentation and analysis of multi-channel cell-based high-throughput screens (HTS), more specifically, dual-channel experiments. Such experiments are used, for example, to measure the phenotype of a pathway-specific reporter gene against a constitutive signal that can be used for normalization purposes. Typical examples for dual-channel experimental setups are dual-luciferase assays, whereby both a firefly and renilla luciferase are measured in the same well. In principle, multiplex assays can consist of many more than two channels, such as in the case of flow-cytometry readout or other microscopy-based high-content approaches.

We note that in this report we present a simple approach to analyse data from dual-channel experiments, which can be expanded to experiments

with more than two reporters, taking the in-built normalization functions of *cellHTS* as a template, and employing the extensive statistical modeling capabilities of the R programming language. Moreover, such analyses should be adapted to the biological system and to the question of interest.

This text has been produced as a reproducible document [1], containing the actual computer instructions, given in the language R, to produce all results, including the figures and tables that are shown here. To reproduce the computations shown here, you will need an installation of R (version 2.3 or greater) together with a recent version of the package *cellHTS* and of some other add-on packages. Then, we can simply take the file *twoChannels.Rnw* in the *doc* directory of the package, open it in a text editor, run it using the R command *Sweave*, and modify it according to your needs.

We start by loading the package.

```
> library("cellHTS")
```

2 Assembling the data

Here, we consider a sample data of a dual-channel experiment performed with *D. melanogaster* cells. The screen was conducted in microtiter plate format using a library of double-stranded RNAs (dsRNAs), in duplicates. The example data set corresponds to three 384-well plates. The purpose of the screen is to find signaling components of a given pathway. In the screen, one reporter (assigned to channel 1, and denoted here by R_1) monitors cell growth and viability, while the other reporter (assigned to channel 2 and denoted here by R_2) is indicative of pathway activity.

2.1 Reading the raw intensity files

The set of available result files and the information about them (which plate, which replicate, which channel) is given in the *plate list file*. The first few lines of the plate list file for this data set are shown in Table 1.

Using the function *readPlateData*, we can read the plate list file and all the intensity files, thereby assembling the data into a single R object that can be used for subsequent analyses. First, we define the path for those files:

```
> experimentName = "DualChannelScreen"
> dataPath = system.file(experimentName, package = "cellHTS")
```

The input files are in the `DualChannelScreen` directory of the *cellHTS* package.

Filename	Plate	Replicate	Channel
RA01D1.TXT	1	1	1
RA01D2.TXT	1	2	1
RA02D1.TXT	2	1	1
RA02D2.TXT	2	2	1
RA03D1.TXT	3	1	1
...	...	...	...

Table 1: Selected lines from the example plate list file `Platelist.txt`.

Batch	Well	Content
1	A01	geneA
1	A02	geneB
1	A03	sample
1	A04	sample
...	...	...

Table 2: Selected lines from the example plate configuration file `Plateconf.txt`.

```
> x = readPlateData("Platelist.txt", name = experimentName, path = dataPath)
```

```
> x
```

```
cellHTS object of name 'DualChannelScreen'
3 plates with 384 wells, 2 replicates, 2 channels. State:
configured normalized     scored  annotated
     FALSE      FALSE      FALSE      FALSE
```

2.2 Annotating the plate results

Next, we annotate the measured data with information on the controls, and flag invalid measurements using the information given in the *plate configuration file* and in the *screen log file*, respectively. Selected lines of these files are shown in Table 2 and Table 3. Morevoer, we also add the information contained in the *screen description file*, which gives a general description of the screen.

```
> confFile = file.path(dataPath, "Plateconf.txt")
> logFile = file.path(dataPath, "Screenlog.txt")
```

Filename	Well	Flag	Comment
RA03D1.TXT	A05	NA	contaminated
RB03D1.TXT	A05	NA	contaminated
...	...	...	...

Table 3: Selected lines from the example screen log file `Screenlog.txt`.

```
> descripFile = file.path(dataPath, "Description.txt")
> x = configure(x, confFile, logFile, descripFile)
```

In this data set, instead of using the default names *pos* and *neg* for positive and negative controls, respectively, we use the name of the gene targeted by the probes in the control wells: *geneA*, *geneB*, *geneC* and *geneD*. This is a more straighforward approach, since not all of these four controls behave as controls for both reporters R_1 and R_2.

Below, we look at the frequency of each well annotation in the example data:

```
> table(x$plateConf$Content)

 geneA  geneB  geneC  geneD sample
     1      1      1      1    380
```

3 Data preprocessing and summarization of replicates

We can take a first look at the data by constructing the HTML quality reports using the *writeReport* function.

As mentioned above, the controls used in the screen are reporter-specific. When calling *writeReport*, we need to specify to the function's arguments `posControls` and `negControls` which are the positive and negative controls for each channel:

```
> negControls = vector("character", length = dim(x$xraw)[4])
> negControls[1] = "(?i)^geneA$"
> negControls[2] = "(?i)^geneA$|^geneB$"
> posControls = vector("character", length = dim(x$xraw)[4])
> posControls[2] = "(?i)^geneC$|^geneD$"
```

In the constitutive channel R_1, there is one negative control, named *geneA*, and no positive controls. In the pathway-specific reporter R_2 there are two different negative controls (*geneA* and *geneB*), and two diffferent positive controls (*geneC* and *geneD*). Each of the arguments `posControls` and `negControls` should be defined as a vector of regular expressions with the same length as the number of channels in `x$xraw`. These arguments will be passed to the *regexpr* function for pattern matching within the well annotation given in `x$wellAnno`.

Finally, we construct the quality report pages for the raw data in a directory called `raw`, in the working directory:

```
> out = writeReport(x, outdir = "raw", posControls = posControls,
+     negControls = negControls)
```

After this function has finished, we can view the index page of the report:

```
> browseURL(out)
```

In this experiment, reporter 1 (R_1) monitors cell viability. Thus, wells with low intensities in R_1 should be masked: these cells are not responding to a specific perturbation of the studied signaling pathway, but show a more unspecific cell viability phenotype. There is no obvious choice for a threshold for the minimum intensity R_1 that we consider still viable; here, we have chosen to set this cut-off as a low quantile (5%) of the overall distribution of corrected intensity values in the R_1 channel of each replicate. However, to be able to use the overall distribution of intensities in the three plates, first we need to remove the plate-to-plate variations. This will be performed by applying plate median scaling to each replicate and channel using the function *normalizePlates*:

```
> x = normalizePlates(x, normalizationMethod = "median")
```

```
> ctoff = apply(x$xnorm[, , , 2], 3, quantile, probs = 0.05, na.rm = TRUE)
```

Figure 1 shows the plate median corrected intensities in R_2 versus R_1 channels, together with the calculated threshold and the positive and negative controls of the pathway-inducible reporter R_2. The wells with intensity values below the calculated threshold are shown in grey and will be set to "NA" in `x$xraw`. The subsequent preprocessing will be applied on this array.

```
> for (r in 1:dim(x$xnorm)[3]) {
+     ind = x$xnorm[, , r, 1] <= ctoff[r]
+     x$xraw[, , r, ][ind] = NA
+ }
```

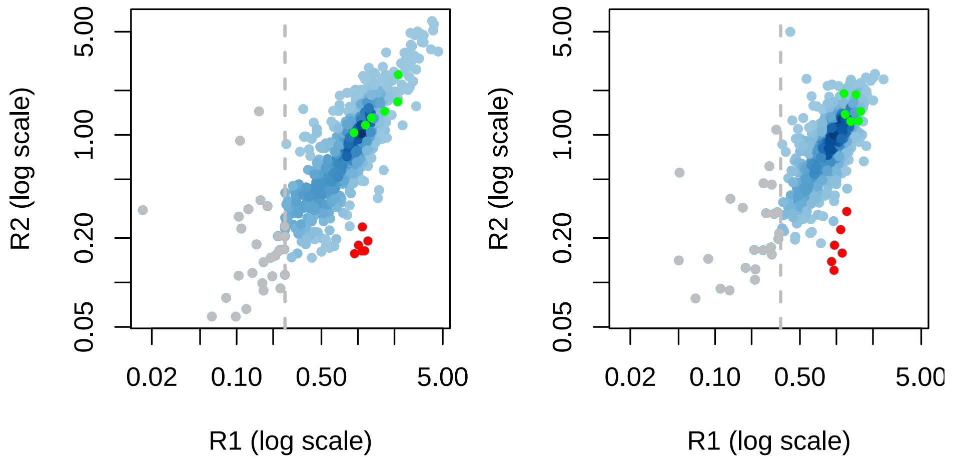

Figure 1: Scatterplot of the plate median corrected intensity values in the signal-dependent channel (R_2) against the plate median corrected intensity values in the constitutive channel (R_1) for replicate 1 (left) and replicate 2 (right). Masked values are shown in grey, while positive and negative controls are shown in red and green, respectively.

In order to distinguish between changes in the readout caused by depletion of specific pathway components versus changes in the overall cell number, the next step consists in normalizing the pathway-inducible readout (R_2) against the constitutive reporter (R_1). This can be done using the *normalizeChannels* function provided in the *cellHTS* package. This function can also adjust for plate effects on the R_2/R_1 ratios (or log ratios) using the method specified in the parameter `adjustPlates`. For example, by setting it to `"median"`, the *normalizeChannels* function applies plate median normalization by dividing or subtracting (if data has been log transformed) each value by the median of values in that plate.

Below, we apply *normalizeChannels* to take the log ratio R_2/R_1 using the data values in `x$xraw`, and then use plate median normalization to remove plate-to-plate variations:

```
> x = normalizeChannels(x, fun = function(r1, r2) r2/r1, log = TRUE,
+     adjustPlates = "median")
```

The normalized intensities are stored in the slot `x$xnorm`. This is an array of the same size as `x$xraw`, except in the last dimension (number of channels).

We can call again *writeReport* for the normalized data. But first, we have to redefine the positive and negative controls for the normalized data `x$xnorm`, because it now corresponds to a single channel. The controls for the normalized data values are the same as those of the raw data channel R_2.

```
> negControls = "(?i)^geneA$|^geneB$"
> posControls = "(?i)^geneC$|^geneD$"
```

```
> out = writeReport(x, outdir = "logRatio", posControls = posControls,
+     negControls = negControls)
```

The quality reports have been created in the folder `logRatio` in the working directory.

Below, we call the *summarizeReplicates* function to determine the z-score values for each replicate, and then summarize the replicated z-score values by taking the average.

```
> x = summarizeReplicates(x, zscore = "-", summary = "mean")
```

The resulting single z-score value per probe is stored in the slot `x$score`. Figure 2 shows the boxplots of the z-scores for the different types of probes.

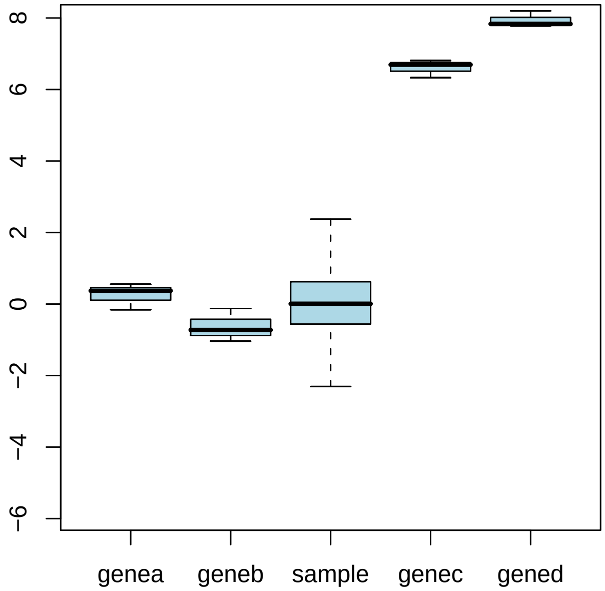

Figure 2: Boxplots of z-scores for the different types of probes.

```
> ylim = quantile(x$score, c(0.001, 0.999), na.rm = TRUE)
> boxplot(x$score ~ x$wellAnno, col = "lightblue", outline = FALSE,
+     ylim = ylim)
```

Now that the data have been preprocessed and scored, we call again *writeReport* and use a web browser to view the resulting report:

```
> out = writeReport(x, force = TRUE, outdir = "logRatio", imageScreenArgs = list(zran
+     4)), plotPlateArgs = list(xrange = c(-3, 3)), posControls = posControls,
+     negControls = negControls)

> browseURL(out)
```

Finally, we will save the data set to a file.

```
> save(x, file = paste(experimentName, ".rda", sep = ""))
```

References

[1] Robert Gentleman. Reproducible research: A bioinformatics case study. *Statistical Applications in Genetics and Molecular Biology*, 3, 2004. 2
