# Supplementary material for: Analysis of cell-based RNAi screens
Source: Genome Biol. 2006 Jul 25;7(7):R66. doi: 10.1186/gb-2006-7-7-r66 (PMC1779553; doi:10.1186/gb-2006-7-7-r66)
Supplement: Additional data file 2 — R package in "Windows binary" format. This file archive also contains the example data. [file gb-2006-7-7-r66-S2.zip › cellHTS/html/00Index.html]

R: Analysis of cell-based screens

# Analysis of cell-based screens

---

## Documentation for package `cellHTS' version 1.3.23

## User Guides and Package Vignettes

Read overview or browse directory.

## Help Pages

|  |  |
| --- | --- |
| annotate | Annotates the gene IDs of a given cellHTS object |
| bdgpbiomart | Dataset with annotation of CG identifiers |
| configure | Configures the plates and plate result files |
| getLibraryPlate | 384-well plate assay format to a 96-well plate library format |
| imageScreen | Experiment-wide quality control plot of a cellHTS object |
| lines.ROC | Creates an object of class "ROC" which can be plotted as a ROC curve |
| normalizeChannels | Normalization of dual-channel data and data transformation |
| normalizePlates | Plate-wise data normalization, and data transformation |
| oneRowPerId | Rearrange dataframe entries such that there is exactly one row per ID. |
| plot.ROC | Creates an object of class "ROC" which can be plotted as a ROC curve |
| plotPlateLibrary | Plate plot of the raw data of the four consecutive 96-well plates of a given 384-well plate |
| print.cellHTS | Printing cellHTS objects |
| readPlateData | Read a collection of plate reader data files |
| ROC | Creates an object of class "ROC" which can be plotted as a ROC curve |
| summarizeReplicates | Summarizes between normalized replicate values given in a cellHTS object, obtaining a single z-score for each probe |
| write.tabdel | Wrapper to function 'write.table' used to write data to a tab-delimited file |
| writeReport | Create a directory with HTML pages of linked tables and plots documenting the contents of a cellHTS object |
| writeTab | Write the data from a cellHTS object to a tab-delimited file |
