# Supplementary material for: Analysis of cell-based RNAi screens
Source: Genome Biol. 2006 Jul 25;7(7):R66. doi: 10.1186/gb-2006-7-7-r66 (PMC1779553; doi:10.1186/gb-2006-7-7-r66)
Supplement: Additional data file 2 — R package in "Windows binary" format. This file archive also contains the example data. [file gb-2006-7-7-r66-S2.zip › cellHTS/html/annotate.html]

R: Annotates the gene IDs of a given cellHTS object

|  |  |
| --- | --- |
| annotate {cellHTS} | R Documentation |

## Annotates the gene IDs of a given cellHTS object

### Description

Annotate the gene IDs of a given cellHTS object.

### Usage

```
annotate(x, ...)
## S3 method for class 'cellHTS':
annotate(x, geneIDFile, ...)
```

### Arguments

|  |  |
| --- | --- |
| `x` | a cellHTS object. |
| `geneIDFile` | the name of the file with the gene IDs (see details). This argument is just passed on to the `read.table` function, so any of the valid argument types for `read.table` are valid here, too. Must contain one row for each well and each plate. |
| `...` | additional parameters - ignored. |

### Details

geneIDFile
:   This file is expected to be a tab-delimited file with at least three columns, and column names `Plate`, `Well` and `GeneID`. The contents of `Plate` are expected to be integer. Further columns are allowed.

### Value

An S3 object of class `cellHTS`, which extends the argument `x` by the following element:

|  |  |
| --- | --- |
| `geneAnno` | a data.frame containing what was read from input file `geneIDFile`. The number of rows is equal to the product between the number of wells in each plate and the number of plates. |

Moreover, the processing status of the `cellHTS` object is updated in the slot `state` to `state["annotated"]= TRUE`.
There are methods `print.cellHTS`, `configure.cellHTS` and `annotate.cellHTS`.

### Author(s)

Wolfgang Huber huber@ebi.ac.uk, Ligia Braz ligia@ebi.ac.uk

### References

..

### Examples

```
 datadir = system.file("KcViabSmall", package = "cellHTS")
 x = readPlateData("Platelist.txt", "KcViabSmall", path=datadir)
 confFile = system.file("KcViabSmall", "Plateconf.txt", package="cellHTS")
 logFile  = system.file("KcViabSmall", "Screenlog.txt", package="cellHTS")
 descripFile  = system.file("KcViabSmall", "Description.txt", package="cellHTS")
 x = configure(x, confFile, logFile, descripFile)
 geneIDFile = system.file("KcViabSmall", "GeneIDs_Dm_HFAsubset_1.0.txt", package="cellHTS")
 x = annotate(x, geneIDFile)
```

---

[Package *cellHTS* version 1.3.23 Index]
