# Supplementary material for: Analysis of cell-based RNAi screens
Source: Genome Biol. 2006 Jul 25;7(7):R66. doi: 10.1186/gb-2006-7-7-r66 (PMC1779553; doi:10.1186/gb-2006-7-7-r66)
Supplement: Additional data file 2 — R package in "Windows binary" format. This file archive also contains the example data. [file gb-2006-7-7-r66-S2.zip › cellHTS/html/bdgpbiomart.html]

R: Dataset with annotation of CG identifiers

|  |  |
| --- | --- |
| bdgpbiomart {cellHTS} | R Documentation |

## Dataset with annotation of CG identifiers

### Description

See the vignette, Section *Using biomaRt to annotate the target
genes online* for details. The annotations were obtained on
12 March 2006.

### Usage

```
data(bdgpbiomart)
```

### Format

Dataframe with 21888 rows and 11 columns
`Plate`, `Well`, `HFAid`, `GeneID`,
`chr_name`, `chrom_start`, `chrom_end`, `description`,
`flybase_name`, `go_id`, `go_description`.

### Source

BioMart webinterface to Ensembl 37.

### Examples

```
data(bdgpbiomart)
```

---

[Package *cellHTS* version 1.3.23 Index]
