# Supplementary material for: Analysis of cell-based RNAi screens
Source: Genome Biol. 2006 Jul 25;7(7):R66. doi: 10.1186/gb-2006-7-7-r66 (PMC1779553; doi:10.1186/gb-2006-7-7-r66)
Supplement: Additional data file 2 — R package in "Windows binary" format. This file archive also contains the example data. [file gb-2006-7-7-r66-S2.zip › cellHTS/html/configure.html]

R: Configures the plates and plate result files

|  |  |
| --- | --- |
| configure {cellHTS} | R Documentation |

## Configures the plates and plate result files

### Description

Annotate the plates and the plate result files of a given cellHTS object.

### Usage

```
configure(x, ...)
## S3 method for class 'cellHTS':
configure(x, confFile, logFile, descripFile, ...)
```

### Arguments

|  |  |
| --- | --- |
| `x` | a cellHTS object. |
| `confFile` | the name of the configuration file (see details). This argument is just passed on to the `read.table` function, so any of the valid argument types for `read.table` are valid here, too. Must contain one row for each well and each batch. |
| `logFile` | optional; the name of the screen log file (see details). This argument is just passed on to the `read.table` function, so any of the valid argument types for `read.table` are valid here, too. |
| `descripFile` | the name of the screen description file (see details). This argument is just passed on to the `readLines` function, so any of the valid argument types for `readLines` are valid here, too. |
| `...` | additional parameters - ignored. |

### Details

confFile
:   This file is expected to be a tab-delimited file with
    at least three columns, and column names `Batch`, `Well` and `Content`. The contents of `Batch` are expected to be integer.

logFile
:   If given as an argument, it is expected to be a tab-delimited file with
    at least three columns, and column names `Filename`, `Well`,
    and `Flag`. Further columns are allowed.

descripFile
:   This file is the screen description file with
    general information about the screen.

Data from wells that are annotated as *empty* are ignored and are set to NA in `x` in slot `xraw`.

### Value

An S3 object of class `cellHTS`, which extends the argument `x` by the following elements:

|  |  |
| --- | --- |
| `plateConf` | a data.frame containing what was read from input file `confFile`. The number of rows is equal to the product between the number of wells in each plate and the number of batches. |
| `screenLog` | a data.frame containing what was read from input file `logFile`. |
| `wellAnno` | object of class `factor` of length number of plates x number of wells per plate, with five possible levels: *empty*, *other*, *neg*, *sample*, and *pos*, indicative of the contents of the wells. |
| `screenDesc` | object of class `character` containing what was read from input file `descripFile`. |

Moreover, the processing status of the `cellHTS` object is updated in the slot `state` to `state["configured"]=TRUE`.
There are methods `print.cellHTS`, `configure.cellHTS` and `annotate.cellHTS`.

### Author(s)

Wolfgang Huber huber@ebi.ac.uk, Ligia Braz ligia@ebi.ac.uk

### References

..

### Examples

```
 datadir = system.file("KcViabSmall", package = "cellHTS")
 x = readPlateData("Platelist.txt", "KcViabSmall", path=datadir)
 confFile = system.file("KcViabSmall", "Plateconf.txt", package="cellHTS")
 logFile  = system.file("KcViabSmall", "Screenlog.txt", package="cellHTS")
 descripFile  = system.file("KcViabSmall", "Description.txt", package="cellHTS")
 x = configure(x, confFile, logFile, descripFile)
```

---

[Package *cellHTS* version 1.3.23 Index]
