# Supplementary material for: Analysis of cell-based RNAi screens
Source: Genome Biol. 2006 Jul 25;7(7):R66. doi: 10.1186/gb-2006-7-7-r66 (PMC1779553; doi:10.1186/gb-2006-7-7-r66)
Supplement: Additional data file 2 — R package in "Windows binary" format. This file archive also contains the example data. [file gb-2006-7-7-r66-S2.zip › cellHTS/html/getLibraryPlate.html]

R: 384-well plate assay format to a 96-well plate library format

|  |  |
| --- | --- |
| getLibraryPlate {cellHTS} | R Documentation |

## 384-well plate assay format to a 96-well plate library format

### Description

Given a cellHTS object with data from an assay conducted in 384-well plate format, resulting from the combination of four consecutive 96-well plates of a reagent library, this function gives the plate identifiers for the 96-well plates.

### Usage

```
getLibraryPlate(x)
```

### Arguments

|  |  |
| --- | --- |
| `x` | a cellHTS object. |

### Details

The cellHTS object `x` contains data from a screening experiment where every set of four consecutive 96-well plates was combined into a 384-well plate. Therefore, the only available plate identifiers are for the assay plate format (384-well plates). The way the four 96-well plates are transferred to a 384-well plate during an experiment is as follows: the robot stars by transferring the samples from the first 96-well plate into the first quadrant of the 384-well plate, and so on.

### Value

An S3 object of class `cellHTS`, which extends the argument `x` by the following element:

|  |  |
| --- | --- |
| `libPlate` | a vector of length equal to the total number of wells of all the 384-well plates, containing a number that identifies the 96-well plate. It ranges from 1 to four times the total number of 384-well plates. |

### Author(s)

Ligia Braz ligia@ebi.ac.uk

### References

..

### Examples

```
 datadir = system.file("KcViabSmall", package = "cellHTS")
 x = readPlateData("Platelist.txt", "KcViabSmall", path=datadir)
 confFile = system.file("KcViabSmall", "Plateconf.txt", package="cellHTS")
 logFile  = system.file("KcViabSmall", "Screenlog.txt", package="cellHTS")
 descripFile  = system.file("KcViabSmall", "Description.txt", package="cellHTS")
 x = configure(x, confFile, logFile, descripFile)
 x = getLibraryPlate(x)
 table(x$libPlate)
```

---

[Package *cellHTS* version 1.3.23 Index]
