# Supplementary material for: Analysis of cell-based RNAi screens
Source: Genome Biol. 2006 Jul 25;7(7):R66. doi: 10.1186/gb-2006-7-7-r66 (PMC1779553; doi:10.1186/gb-2006-7-7-r66)
Supplement: Additional data file 2 — R package in "Windows binary" format. This file archive also contains the example data. [file gb-2006-7-7-r66-S2.zip › cellHTS/html/imageScreen.html]

R: Experiment-wide quality control plot of a cellHTS object

|  |  |
| --- | --- |
| imageScreen {cellHTS} | R Documentation |

## Experiment-wide quality control plot of a cellHTS object

### Description

Experiment-wide quality control plot of a scored cellHTS object.

### Usage

```
imageScreen(x, ar=3/5, zrange)
```

### Arguments

|  |  |
| --- | --- |
| `x` | a cellHTS object that has already been scored (i.e. containg the slot `score`). |
| `ar` | the desired aspect ration for the image plot (i.e. number of columns per number of rows) |
| `zrange` | the range of values to be mapped into the color scale. If missing, zrange will be set to the range of `x$score`. |

### Details

This function creates an image plot that gives an overview of the whole set of score values
from the cellHTS object `x`.

### Author(s)

Ligia Braz ligia@ebi.ac.uk

### Examples

```
 datadir = system.file("KcViabSmall", package = "cellHTS")
 x = readPlateData("Platelist.txt", "KcViabilitySmall", path=datadir)
 confFile = system.file("KcViabSmall", "Plateconf.txt", package="cellHTS")
 logFile  = system.file("KcViabSmall", "Screenlog.txt", package="cellHTS")
 descripFile  = system.file("KcViabSmall", "Description.txt", package="cellHTS")
 x = configure(x, confFile, logFile, descripFile)
 x = normalizePlates(x, normalizationMethod="median",zscore="-")
 x = summarizeReplicates(x) 
 imageScreen(x, zrange=c(-5,5))
```

---

[Package *cellHTS* version 1.3.23 Index]
