# Supplementary material for: Analysis of cell-based RNAi screens
Source: Genome Biol. 2006 Jul 25;7(7):R66. doi: 10.1186/gb-2006-7-7-r66 (PMC1779553; doi:10.1186/gb-2006-7-7-r66)
Supplement: Additional data file 2 — R package in "Windows binary" format. This file archive also contains the example data. [file gb-2006-7-7-r66-S2.zip › cellHTS/html/normalizeChannels.html]

R: Normalization of dual-channel data and data transformation

|  |  |
| --- | --- |
| normalizeChannels {cellHTS} | R Documentation |

## Normalization of dual-channel data and data transformation

### Description

Normalizes and/or transforms dual-channel data `xraw` of a given 'cellHTS' object by applying the function defined in `fun`. The default is to take the ratio between the second and first channels (R2/R1).
Correction of plate-to-plate variations may also be performed.

### Usage

```
normalizeChannels(x, fun=function(r1,r2) r2/r1, log=FALSE, adjustPlates, zscore)
```

### Arguments

|  |  |
| --- | --- |
| `x` | a cellHTS object that has already been configured (see details). |
| `fun` | a function defined by the user to relate the signal in the two channels `r1` and `r2`. `fun` takes two numeric vectors and returns a numeric vector of the same length. The default is to take the ration between the second and first channels. |
| `log` | a logical value indicating whether the result obtained after applying `fun` should be log2 transformed. The default is `log = FALSE`, and the data is not log2 transformed. |
| `adjustPlates` | character string indicating the correction factor to apply to adjust for plate-to-plate varations, after applying `fun` and eventually log transforming the values (see details). If `adjustPlates` is missing (the default), no plate-wise correction will be performed. |
| `zscore` | indicates if the z-scores should be determined after normalization and transformation. If missing (default), the data will not be scored. Otherwise, it should be a character string, either "+" or "-", specifying the sign to use for the calculated z-scores (see details). |

### Details

For each plate and replicate of a two-color experiment, the function
defined in `fun` is applied to relate the intensity values in the
two channels of the `cellHTS` object. The default is to calculate
the ratio between the second and the first channels, but other options can be defined.

If `log = TRUE`, the data obtained after applying `fun` is
log2 transformed. The default is `log = FALSE`.

If `adjustPlates` is not missing, the obtained values will be further corrected for plate effects by considering the chosen per-plate factor. If `adjustPlates="mean"`, this factor will be the plate mean, if `adjustPlates="median"`, the plate median, whereas if `adjustPlates="shorth"`, the midpoint of the shorth (see `shorth`) of the distribution of values. In either case, the median, mean or midpoint of the shorth is determined for the distribution of values in the wells annotated as `sample` in `x$wellAnno`, for a given replicated plate. If the data have not been log transformed (`log=FALSE`), each measurement is divided by the per-plate plate factor, while if the data values are in log2 scale (`log=TRUE`), the per-plate factor will be subtracted from each measurement. By default, `adjustPlates` is missing.

If `zscore` is not missing, a robust z-score for each
individual measurement will be determined for each plate and each well
by subtracting the overall median and dividing by the overall mad. The
overall median and mad are taken by considering the distribution of
intensities (over all plates) in the wells whose content is annotated as
`sample`.
The allowed values for `zscore` ("+" or "-") are used to set the sign of
the calculated z-scores. For example, with a `zscore="-"` a strong decrease
in the signal will be represented by a positive z-score, whereas setting `zscore="+"`,
such a phenotype will be represented by a negative z-score.
This option can be set to calculate the results to the commonly used convention.

### Value

An object of class `cellHTS`, which is a copy of the
argument `x`, plus an additional slot `xnorm` containing the
normalized data. This is an array of the same dimensions as `xraw`,
except in the dimension corresponding to the number of channels, since
the two-channel intensities have been combined into one intensity value.
  
Moreover, the processing status of the `cellHTS` object is updated
in the slot `state` to `state["normalized"]=TRUE`.

### Author(s)

Ligia Braz ligia@ebi.ac.uk, Wolfgang Huber huber@ebi.ac.uk

### References

..

### Examples

```
datadir = system.file("DualChannelScreen", package = "cellHTS")
x = readPlateData("Platelist.txt", "TwoColorData", path=datadir)
confFile = system.file("DualChannelScreen", "Plateconf.txt", package="cellHTS")
logFile  = system.file("DualChannelScreen", "Screenlog.txt", package="cellHTS")
descripFile  = system.file("DualChannelScreen", "Description.txt", package="cellHTS")
x = configure(x, confFile, logFile, descripFile)

table(x$wellAnno)

## Define the controls for the different channels:
negControls=vector("character", length=dim(x$xraw)[4])

 # channel 1 - gene A
 negControls[1]= "(?i)^geneA$" # case-insensitive and match the empty string at the beginning and end of a line (to distinguish between "geneA" and "geneAB", for example. Although it is not a problem for the present well annotation)
 
 # channel 2 - gene A and geneB
 negControls[2]= "(?i)^geneA$|^geneB$" 

posControls = vector("character", length=dim(x$xraw)[4])
 # channel 1 - no controls
 # channel 2 - geneC and geneD
 posControls[2]="(?i)^geneC$|^geneD$"

#writeReport(x, posControls=posControls, negControls=negControls)
x = normalizeChannels(x, fun=function(x,y) y/x, log=TRUE, adjustPlates="median")

## Define the controls for the normalized intensities (only one channel):
negControls = vector("character", length=dim(x$xnorm)[4])
# For the single channel, the negative controls are geneA and geneB 
negControls[1]= "(?i)^geneA$|^geneB$" 

posControls = vector("character", length=dim(x$xnorm)[4])
# For the single channel, the negative controls are geneC and geneD 
posControls[1]="(?i)^geneC$|^geneD$"
#writeReport(x, force=TRUE, plotPlateArgs=list(xrange=c(-3,3)), posControls=posControls, negControls=negControls)
```

---

[Package *cellHTS* version 1.3.23 Index]
