# Supplementary material for: Analysis of cell-based RNAi screens
Source: Genome Biol. 2006 Jul 25;7(7):R66. doi: 10.1186/gb-2006-7-7-r66 (PMC1779553; doi:10.1186/gb-2006-7-7-r66)
Supplement: Additional data file 2 — R package in "Windows binary" format. This file archive also contains the example data. [file gb-2006-7-7-r66-S2.zip › cellHTS/html/normalizePlates.html]

R: Plate-wise data normalization, and data transformation

|  |  |
| --- | --- |
| normalizePlates {cellHTS} | R Documentation |

## Plate-wise data normalization, and data transformation

### Description

Per-plate normalization of the data `xraw` of a cellHTS object.
Optionally, a data transformation such as `log`, and a
transformation to z-scores can be performed.

### Usage

```
normalizePlates(x, normalizationMethod="median", transform, zscore, posControls, negControls)
```

### Arguments

|  |  |
| --- | --- |
| `x` | a `cellHTS` object that has already been configured (see details). |
| `normalizationMethod` | a character specifying the normalization method to use for performing the per-plate normalization. Allowed values are `"median"` (default), `"mean"`, `"shorth"`, `"POC"` and `"NPI"` (see details). |
| `transform` | a function that takes a numeric vector and returns a numeric vector of the same length; for example, the logarithm function `log`. |
| `zscore` | indicates if the data should be centered and scaled after normalization and transformation. If missing (default), the data will not be centered and scaled. Otherwise, the value of this argument should be a character string, either "+" or "-", which will be used to set the sign for the calculated z-scores (see details). |
| `posControls` | a vector of regular expressions giving the name of the positive control(s). See details. |
| `negControls` | a vector of regular expressions giving the name of the negative control(s). See details. |

### Details

The normalization is performed in a per-plate fashion.
If `normalizationMethod="median"`, plates effects are corrected by dividing each measurement
by the median value across wells annotated as `sample` in `x$wellAnno`, for each plate and replicate.
If `normalizationMethod="mean"`, the average in the `sample` wells is consider instead.
If `normalizationMethod="shorth"`, for each plate and replicate, the midpoint of the shorth (see `shorth`) of the distribution of values in the wells annotated as `sample` is
calculated. Then, every measurement is divided by this value.
If `normalizationMethod="POC"`, for each plate and replicate, each measurement is divided by the average of the measurements on the plate positive controls.
If `normalizationMethod="NPI"`, each measurement is subtracted from the average of the intensities on the plate positive controls, and this result is divided by the difference between the means of the measurements on the positive and the negative controls.

If `transform` is not missing, the chosen data transformation is applied.
Most commonly, this option can be used to apply a log transformation.

If `zscore` is not missing, a robust z-score for each individual
measurement will be determined for each plate and each well by
subtracting the overall `median` and dividing by the overall
`mad`. These are taken by considering
the distribution of intensities (over all plates) in the wells whose
content is annotated as `sample`. The allowed values for
`zscore` ("+" or "-") are used to set the sign of the calculated z-scores.
For example, with a `zscore="-"` a strong decrease in the signal will be represented by a positive z-score, whereas setting `zscore="+"`,
such a phenotype will be represented by a negative z-score.
This option can be set to calculate the results to the commonly used convention.

The arguments `posControls` and `negControls` are required for applying the normalization methods based on the control measurements (that is, when `normalizationMethod="POC"` or `normalizationMethod="NPI"`).
`posControls` and `negControls` should be given as a vector of regular expression patterns specifying the name of the positive(s) and negative(s) controls, respectivey, as provided in the plate configuration file (and stored in `x$wellAnno`). The length of these vectors should be equal to the number of reporters used in the screen (`dim(x$xraw)[4]`) or to `dim(x$xnorm)[4]`, in case 'x' contains multi-channel data that has been normalized by combining the values from two or more channels.
By default, if `posControls` is not given, "pos" will be taken as the name for the wells containing positive controls. Similarly, if `negControls` is missing, by default "neg" will be considered as the name used to annotated the negative controls.
The content of `posControls` and `negControls` will be passed to `regexpr` for pattern matching within the well annotation given in `x$wellAnno` (see examples).
The arguments `posControls` and `negControls` are particularly useful in multi-channel data since the controls might be reporter-specific, or after normalizing multi-channel data.

### Value

An object of class `cellHTS`, which is a copy of the argument
`x`, plus an additional slot `xnorm` containing the normalized
data. This is an array of the same dimensions as `xraw`.
  
Moreover, the processing status of the `cellHTS` object is updated
in the slot `state` to `state["normalized"]=TRUE`.

### Author(s)

Ligia Braz ligia@ebi.ac.uk, Wolfgang Huber huber@ebi.ac.uk

### References

..

### Examples

```
 datadir = system.file("KcViabSmall", package = "cellHTS")
 x = readPlateData("Platelist.txt", "KcViabSmall", path=datadir)
 confFile = system.file("KcViabSmall", "Plateconf.txt", package="cellHTS")
 logFile  = system.file("KcViabSmall", "Screenlog.txt", package="cellHTS")
 descripFile  = system.file("KcViabSmall", "Description.txt", package="cellHTS")
 x = configure(x, confFile, logFile, descripFile)
 x = normalizePlates(x, normalizationMethod="median", zscore="-")
```

---

[Package *cellHTS* version 1.3.23 Index]
