# Supplementary material for: Analysis of cell-based RNAi screens
Source: Genome Biol. 2006 Jul 25;7(7):R66. doi: 10.1186/gb-2006-7-7-r66 (PMC1779553; doi:10.1186/gb-2006-7-7-r66)
Supplement: Additional data file 2 — R package in "Windows binary" format. This file archive also contains the example data. [file gb-2006-7-7-r66-S2.zip › cellHTS/html/oneRowPerId.html]

R: Rearrange dataframe entries such that there is exactly one row
per ID.

|  |  |
| --- | --- |
| oneRowPerId {cellHTS} | R Documentation |

## Rearrange dataframe entries such that there is exactly one row per ID.

### Description

Rearrange dataframe entries such that there is exactly one row
per ID. The IDs are taken from the argument `ids` and are matched
against the first column of `x`. If an ID is missing in `x[,1]`,
a row with `NA` values is inserted. If an ID occurs multiple times
in `x[,1]`, rows are collapsed into characters of comma-separated values.

### Usage

```
oneRowPerId(x, ids)
```

### Arguments

|  |  |
| --- | --- |
| `x` | dataframe. |
| `ids` | character vector. |

### Details

### Value

A dataframe whose rows correspond 1:1 to `ids`.

### Author(s)

W. Huber huber@ebi.ac.uk, Ligia Pedroso Bras ligia@ebi.ac.uk

### Examples

```
x = data.frame(ids=I(c("a", "a", "c")), val=11:13)
oneRowPerId(x, letters[1:3])
```

---

[Package *cellHTS* version 1.3.23 Index]
