# Supplementary material for: Analysis of cell-based RNAi screens
Source: Genome Biol. 2006 Jul 25;7(7):R66. doi: 10.1186/gb-2006-7-7-r66 (PMC1779553; doi:10.1186/gb-2006-7-7-r66)
Supplement: Additional data file 2 — R package in "Windows binary" format. This file archive also contains the example data. [file gb-2006-7-7-r66-S2.zip › cellHTS/html/plotPlateLibrary.html]

R: Plate plot of the raw data of the four consecutive 96-well plates of a given 384-well plate

|  |  |
| --- | --- |
| plotPlateLibrary {cellHTS} | R Documentation |

## Plate plot of the raw data of the four consecutive 96-well plates of a given 384-well plate

### Description

Given a cellHTS object with data from an assay where every set of four consecutive 96-well plates was combined into a 384-well plate, this function plots the raw intensities of a chosen 384-well assay plate distributed according to the 96-well plate format.

### Usage

```
plotPlateLibrary(x, whichPlate=1, whichChannel=1, plotSd=TRUE, plotPlateArgs)
```

### Arguments

|  |  |
| --- | --- |
| `x` | a cellHTS object containing the slot `libPlate` with the 96-well plate identifiers (see `getLibraryPlate`). |
| `whichPlate` | a number indicating the 384-well plate that we want to examine. (By default, it considers the first plate, `whichPlate=1`). |
| `whichChannel` | a number indicating the channel that we want to consider. (By default, the first channel is considered: `whichChannel=1`) |
| `plotSd` | a logical value indicating whether the standard deviation across replicates should be plotted (default is `plotSd=TRUE`. |
| `plotPlateArgs` | optional argument. If given, should be a list with parameters for the plate plots. See details. |

### Details

The cellHTS object `x` contains data from a screening experiment where every set of four consecutive 96-well plates was combined into a 384-well plate. The plate identifiers for the 96-well plates are given in the slot `libPlate`, obtained by `getLibraryPlate`.

Given the channel specified by `whichChannel` and the 384-well plate number `whichPlate`, the function plots the raw intensities for each replicate in both the 96-well and the 384-well plate format. If `plotSd=TRUE`, the standard deviation across replicates is also plotted.

The following elements are recognized for `plotPlateArgs` and
passed on to `plotPlate`:
`sdcol`, the color scheme for the standard deviation plate plot,
`sdrange`, the sd range to which the colors are mapped,
`xcol`, the color scheme for the intensity plate plot,
`xrange`, the intensity range to which the colors are mapped.
If an element is not specified, default values are used.

### Author(s)

Ligia Braz ligia@ebi.ac.uk

### References

..

### See Also

`plotPlate`,
`getLibraryPlate`

### Examples

```
 datadir = system.file("KcViabSmall", package = "cellHTS")
 x = readPlateData("Platelist.txt", "KcViabSmall", path=datadir)
 confFile = system.file("KcViabSmall", "Plateconf.txt", package="cellHTS")
 logFile  = system.file("KcViabSmall", "Screenlog.txt", package="cellHTS")
 descripFile  = system.file("KcViabSmall", "Description.txt", package="cellHTS")
 x = configure(x, confFile, logFile, descripFile)
 x = getLibraryPlate(x) 
 plotPlateLibrary(x, whichPlate=2, plotSd=TRUE)
```

---

[Package *cellHTS* version 1.3.23 Index]
