# Supplementary material for: Analysis of cell-based RNAi screens
Source: Genome Biol. 2006 Jul 25;7(7):R66. doi: 10.1186/gb-2006-7-7-r66 (PMC1779553; doi:10.1186/gb-2006-7-7-r66)
Supplement: Additional data file 2 — R package in "Windows binary" format. This file archive also contains the example data. [file gb-2006-7-7-r66-S2.zip › cellHTS/html/print.cellHTS.html]

R: Printing cellHTS objects

|  |  |
| --- | --- |
| print.cellHTS {cellHTS} | R Documentation |

## Printing cellHTS objects

### Description

Print an object of the class 'cellHTS'.

### Usage

```
## S3 method for class 'cellHTS':
print(x, ...)
```

### Arguments

|  |  |
| --- | --- |
| `x` | object of class `cellHTS`. |
| `...` | optional arguments to `print` methods. |

### Details

Shows the information about the cellHTS object `x`, namely, its name, state, and the number of plates, wells, replicates and channels.

### Author(s)

Wolfgang Huber huber@ebi.ac.uk, Ligia Braz ligia@ebi.ac.uk

### Examples

```
 datadir = system.file("KcViabSmall", package = "cellHTS")
 x = readPlateData("Platelist.txt", "KcViabSmall", path=datadir)
 print(x)
 confFile = system.file("KcViabSmall", "Plateconf.txt", package="cellHTS")
 logFile  = system.file("KcViabSmall", "Screenlog.txt", package="cellHTS")
 descripFile  = system.file("KcViabSmall", "Description.txt", package="cellHTS")
 x = configure(x, confFile, logFile, descripFile)
 print(x)
 geneIDFile = system.file("KcViabSmall", "GeneIDs_Dm_HFAsubset_1.0.txt", package="cellHTS")
 x = annotate(x, geneIDFile)
 print(x)
```

---

[Package *cellHTS* version 1.3.23 Index]
