# Supplementary material for: Analysis of cell-based RNAi screens
Source: Genome Biol. 2006 Jul 25;7(7):R66. doi: 10.1186/gb-2006-7-7-r66 (PMC1779553; doi:10.1186/gb-2006-7-7-r66)
Supplement: Additional data file 2 — R package in "Windows binary" format. This file archive also contains the example data. [file gb-2006-7-7-r66-S2.zip › cellHTS/html/readPlateData.html]

R: Read a collection of plate reader data files

|  |  |
| --- | --- |
| readPlateData {cellHTS} | R Documentation |

## Read a collection of plate reader data files

### Description

Reads a collection of plate reader data files into a data.frame.
The names of the files, plus additional information
(plate number, repeat number) is expected in a tab-delimited table specified
by the argument 'x'.

### Usage

```
readPlateData(filename, path=dirname(filename), name, importFun, verbose=TRUE, plateType)
```

### Arguments

|  |  |
| --- | --- |
| `filename` | the name of the file table (see details). This argument is just passed on to the `read.table` function, so any of the valid argument types for `read.table` are valid here, too. |
| `name` | a character of length 1 with the experiment name. |
| `path` | a character of length 1 indicating the path in which to find the plate reader files. By default, it can extract the path from `filename`. |
| `importFun` | a function that should be used to read each plate result file. The default function works for plate reader data files. See details. |
| `verbose` | a logical value, if TRUE, the function reports some of its intermediate progress. |
| `plateType` | (deprecated argument) a character of length 1 giving the format of the plate: "96" for 96-well plate format, or "384" for a 384-well plate format. |

### Details

The file table is expected to be a tab-delimited file with at
least three columns, and column names `Filename`, `Plate`,
and `Replicate`. The contents of the columns `Plate` and
`Replicate` are expected to be integers. Further columns are
allowed.

We distinguish between *plates* and *plate result file*.
A plate result file contains the measurements results for all
replicates and all channels of a plate, which is the physical carrier
of the reagents.

`importFun` can be used to define other functions to import other data files, such as flow cytometry data files, etc. The `importFun` function should receive as an input the name of a result plate file to read, and return a list with two components. The first component should be a 'data.frame' with the following slots:

well
:   a character vector with the well identifier in the plate.

val
:   the intensity values measured at each well.

The second component of this list can be of any type. The idea is that it contains a copy of the imported input data file.

### Value

An object of class `"cellHTS"`, which is currently implemented as
a list with elements

|  |  |
| --- | --- |
| `name` | copy of the input argument `name` |
| `xraw` | an array of dimension plateSize x number of plates x number of replicates x number of channels, containg the imported measurement data. |
| `pdim` | a numeric vector of length 2 containg the number of rows and columns in a plate. The product of these two numbers is the first dimension of `xraw`. This corresponds to the plate format used in the screen, and it is automatically determined from the plate result files. The allowed formats are 96-well or 384-well plates. |
| `batch` | an integer vector with the batch number (1, 2, ...) for each plate. Its length corresponds to the second dimension of of `xraw`. |
| `plateList` | a data.frame containing what was read from input file `x`, plus a column `status` of type character: it contains the string "OK" if the data import appeared to have gone well, and the respective error or warning message otherwise. |
| `intensityFiles` | a list, where each component contains a copy of the imported input data files. Its length corresponds to the number of rows of `plateList`. |
| `state` | a logical vector representing the processing status of the object. |

### Author(s)

W. Huber huber@ebi.ac.uk

### References

..

### Examples

```
 datadir = system.file("KcViabSmall", package = "cellHTS")
 x = readPlateData("Platelist.txt", "KcViabSmall", path=datadir)
```

---

[Package *cellHTS* version 1.3.23 Index]
