# Supplementary material for: Analysis of cell-based RNAi screens
Source: Genome Biol. 2006 Jul 25;7(7):R66. doi: 10.1186/gb-2006-7-7-r66 (PMC1779553; doi:10.1186/gb-2006-7-7-r66)
Supplement: Additional data file 2 — R package in "Windows binary" format. This file archive also contains the example data. [file gb-2006-7-7-r66-S2.zip › cellHTS/html/ROC.html]

R: Creates an object of class "ROC" which can be plotted as a ROC curve

|  |  |
| --- | --- |
| ROC {cellHTS} | R Documentation |

## Creates an object of class "ROC" which can be plotted as a ROC curve

### Description

The function `ROC` construct an object of S3 class `ROC`,
which represents a receiver-operator-characteristic curve,
from the data of the annotated positive and negative controls in a
scored cellHTS object.

### Usage

```
ROC(x, positives="pos", negatives = "neg")
## S3 method for class 'ROC':
plot(x, col="darkblue", type="l", ...)
## S3 method for class 'ROC':
lines(x, ...)
```

### Arguments

|  |  |
| --- | --- |
| `x` | a cellHTS object that has already been scored (see details). |
| `positives` | a character giving the name(s) used to annotate the positive controls in the cellHTS object. |
| `negatives` | a character corresponding to the name(s) used to annotate the negative controls in the cellHTS object. |
| `col` | the graphical parameter for color; see `par` for details. |
| `type` | the graphical parameter giving the type of plot desired; see `par` for details. |
| `...` | other graphical parameters as in `par` may be also passed as arguments. |

### Details

The `cellHTS` object `x` must contain a slot called `score`,
and selection proceeds from large to small values of this score.
Furthermore, `x` is expected to contain positive and negative
controls annotated in the slot `wellAnno` with the values of
the arguments `positives` and `negatives`, respectively.

### Value

An S3 object of class `ROC`. There are methods
`plot.ROC` and `lines.ROC`.

### Author(s)

W. Huber huber@ebi.ac.uk

### Examples

```
 datadir = system.file("KcViabSmall", package = "cellHTS")
 x = readPlateData("Platelist.txt", "KcViabilitySmall", path=datadir)
 confFile = system.file("KcViabSmall", "Plateconf.txt", package="cellHTS")
 logFile  = system.file("KcViabSmall", "Screenlog.txt", package="cellHTS")
 descripFile  = system.file("KcViabSmall", "Description.txt", package="cellHTS")
 x = configure(x, confFile, logFile, descripFile)
 x = normalizePlates(x, normalizationMethod="median",zscore="-")
 x = summarizeReplicates(x)
 y = ROC(x)
 plot(y)
 lines(y)
```

---

[Package *cellHTS* version 1.3.23 Index]
