# Supplementary material for: Analysis of cell-based RNAi screens
Source: Genome Biol. 2006 Jul 25;7(7):R66. doi: 10.1186/gb-2006-7-7-r66 (PMC1779553; doi:10.1186/gb-2006-7-7-r66)
Supplement: Additional data file 2 — R package in "Windows binary" format. This file archive also contains the example data. [file gb-2006-7-7-r66-S2.zip › cellHTS/html/summarizeReplicates.html]

R: Summarizes between normalized replicate values given in a cellHTS object, obtaining a single z-score for each probe

|  |  |
| --- | --- |
| summarizeReplicates {cellHTS} | R Documentation |

## Summarizes between normalized replicate values given in a cellHTS object, obtaining a single z-score for each probe

### Description

Summarizes the normalized (and possibly already scored) replicate values given in a cellHTS object, and calculates a single z-score value for each probe.

Currently this function is implemented only for single-color data.

### Usage

```
summarizeReplicates(x, zscore, summary="min")
```

### Arguments

|  |  |
| --- | --- |
| `x` | a cellHTS object that has already been normalized (see details). |
| `zscore` | indicates if the replicate values should be centered and scaled. If missing (default), the data will not be centered and scaled. Otherwise, the value of this argument should be a character string, either "+" or "-", specifying the sign to use for the calculated z-scores (see details). |
| `summary` | a character string indicating how to summarize between replicated measurements. One of "min" (default), "mean", "min", or "rms" can be used (see details). |

### Details

Given the normalized values given in the slot `xnorm` of `x`, a single z-score is calculated for each probe.

The argument `zscore` indicates the state of the normalized replicate measurements: if `zscore` is missing, it
is assumed that the replicates have been scored, by calling `normalizePlates`
with the argument `zscore` equal to "-" or "+"; Otherwise, `zscore` should be given, so that a robust z-score
is calculated for each plate and each well by subtracting the overall median and dividing by the overall mad. The overall median and
mad are taken by considering the distribution of intensities (over all plates) in the wells whose content is annotated as `sample`.
The allowed values for `zscore` ("+" or "-") are used to set the sign of
the calculated z-scores. For example, with a `zscore="-"` a strong decrease in the signal will be represented
by a positive z-score, whereas setting `zscore="+"`,
such a phenotype will be represented by a negative z-score.
This option can be set to calculate the results to the commonly used convention.

Finally, a single z-score per probe is calculated by summarizing between scored replicates. If `summary="mean"`,
the average of replicate values is considered; if
`summary="max"`, then the maximum of replicate
intensities is taken; if `summary="min"`, the minimum is considered, instead (conservative); if `summary="rms"`, the
square root of the mean squared value of the replicates (root mean square) is taken as a summary function.

### Value

An object of class `cellHTS`, which is a copy of the argument
`x` plus the slot `score`,
a numeric vector containing the z-factor for each well in
every plate. The length of this vector is therefore equal to the product
between the plateSize and the number of plates.
Moreover, the processing status of the `cellHTS` object is updated
in the slot `state` to `state["scored"]= TRUE`.

### Author(s)

W. Huber huber@ebi.ac.uk, Ligia Braz ligia@ebi.ac.uk

### References

..

### Examples

```
 datadir = system.file("KcViabSmall", package = "cellHTS")
 x = readPlateData("Platelist.txt", "KcViabSmall", path=datadir)
 confFile = system.file("KcViabSmall", "Plateconf.txt", package="cellHTS")
 logFile  = system.file("KcViabSmall", "Screenlog.txt", package="cellHTS")
 descripFile  = system.file("KcViabSmall", "Description.txt", package="cellHTS")
 x = configure(x, confFile, logFile, descripFile)
 x = normalizePlates(x, normalizationMethod="median")
 x = summarizeReplicates(x, zscore="-", summary="min")
```

---

[Package *cellHTS* version 1.3.23 Index]
