# Supplementary material for: Analysis of cell-based RNAi screens
Source: Genome Biol. 2006 Jul 25;7(7):R66. doi: 10.1186/gb-2006-7-7-r66 (PMC1779553; doi:10.1186/gb-2006-7-7-r66)
Supplement: Additional data file 2 — R package in "Windows binary" format. This file archive also contains the example data. [file gb-2006-7-7-r66-S2.zip › cellHTS/html/write.tabdel.html]

R: Wrapper to function 'write.table' used to write data to a tab-delimited file

|  |  |
| --- | --- |
| write.tabdel {cellHTS} | R Documentation |

## Wrapper to function 'write.table' used to write data to a tab-delimited file

### Description

Wrapper for the function `write.table`
to write data to a tab-delimited file.

### Usage

```
write.tabdel(...)
```

### Arguments

|  |  |
| --- | --- |
| `...` | arguments that get passed on to the function `write.table`. |

### Details

A trivial function, which we have included for convenience.

### Value

The name of the file that was written.

### Author(s)

Ligia Braz ligia@ebi.ac.uk

### See Also

`write.table`

### Examples

```
 datadir = system.file("KcViabSmall", package = "cellHTS")
 x = readPlateData("Platelist.txt", "KcViabSmall", path=datadir)
 confFile = system.file("KcViabSmall", "Plateconf.txt", package="cellHTS")
 logFile  = system.file("KcViabSmall", "Screenlog.txt", package="cellHTS")
 descripFile  = system.file("KcViabSmall", "Description.txt", package="cellHTS")
 x = configure(x, confFile, logFile, descripFile)
 geneIDFile = system.file("KcViabSmall", "GeneIDs_Dm_HFAsubset_1.0.txt", package="cellHTS")
 x = annotate(x, geneIDFile)

 # determine the ratio between each well and the plate median
 y = array(as.numeric(NA), dim=dim(x$xraw))
 nrWell = dim(x$xraw)[1]
 for(p in 1:(dim(x$xraw)[2])) {
    samples = (x$wellAnno[(1:nrWell)+nrWell*(p-1)]=="sample")
    y[, p, , ] = apply(x$xraw[, p, , , drop=FALSE], 3:4, function(w) w/median(w[samples], na.rm=TRUE))
 }
 y=signif(y, 4)
 out = matrix(y, nrow=prod(dim(y)[1:2]), ncol=dim(y)[3:4])
 out = cbind(x$geneAnno, x$wellAnno, out)
 colnames(out) = c(names(x$geneAnno), "wellAnno",
  sprintf("Well/Median_r%d_ch%d", rep(1:dim(y)[3], dim(y)[4]), rep(1:dim(y)[4], each=dim(y)[3])))
 write.tabdel(out, file=tempfile())
```

---

[Package *cellHTS* version 1.3.23 Index]
