# Supplementary material for: Analysis of cell-based RNAi screens
Source: Genome Biol. 2006 Jul 25;7(7):R66. doi: 10.1186/gb-2006-7-7-r66 (PMC1779553; doi:10.1186/gb-2006-7-7-r66)
Supplement: Additional data file 2 — R package in "Windows binary" format. This file archive also contains the example data. [file gb-2006-7-7-r66-S2.zip › cellHTS/html/writeReport.html]

R: Create a directory with HTML pages of linked tables and plots
documenting the contents of a cellHTS object

|  |  |
| --- | --- |
| writeReport {cellHTS} | R Documentation |

## Create a directory with HTML pages of linked tables and plots documenting the contents of a cellHTS object

### Description

Creates a directory with HTML pages of linked tables and plots
documenting the contents of a cellHTS object.

### Usage

```
writeReport(x, outdir=file.path(getwd(), x$name),
  force=FALSE, plotPlateArgs=FALSE, imageScreenArgs=NULL, posControls, negControls)
```

### Arguments

|  |  |
| --- | --- |
| `x` | a cell HTS object. |
| `outdir` | a character of length 1 with the name of a directory where to write the report HTML file and images. If the directory does not exist, it is created. If it exists and is not empty, then the behaviour depends on the value of `force`. |
| `force` | a logical value, determines the behaviour of the function if `outdir` exists and is not empty. If `force` is `TRUE`, the function overwrites (removes and recreates) `outdir`, otherwise it casts an error. |
| `plotPlateArgs` | either a list with parameters for the plate plots the per plate quality report pages, or a logical with value `FALSE`. If `FALSE`, the plate plots are omitted, this option is here because the production of the plate plots takes a long time. See details. |
| `imageScreenArgs` | a list with parameters for the function `imageScreen`. See details. |
| `posControls` | a vector of regular expressions specifying the name of the positive controls. See details. |
| `negControls` | a vector of regular expressions specifying the name of the negative controls. See details. |

### Details

The following elements are recognized for `plotPlateArgs` and
passed on to `plotPlate`:
`sdcol`, the color scheme for the standard deviation plate plot,
`sdrange`, the sd range to which the colors are mapped,
`xcol`, the color scheme for the intensity plate plot,
`xrange`, the intensity range to which the colors are mapped.
If an element is not specified, default values are used.

The following elements are recognized for `imageScreenArgs` and
passed on to `imageScreen`:
`ar`, aspect ratio,
`zrange`, range.

`posControls` and `negControls` should be given as a vector of regular expression patterns specifying the name of the positive(s) and negative(s) controls, respectivey, as provided in the plate configuration file (and stored in `x$wellAnno`). The length of these vectors should be equal to the number of reporters used in the screen (`dim(x$xraw)[4]`) or to `dim(x$xnorm)[4]`, in case 'x' contains multi-channel data that has been normalized by combining the values from two or more channels.
By default, if `posControls` is not given, "pos" will be taken as the name for the wells containing positive controls. Similarly, if `negControls` is missing, by default "neg" will be considered as the name used to annotated the negative controls.
The content of `posControls` and `negControls` will be
passed to `regexpr` for pattern matching
within the well annotation given in `x$wellAnno` (see
examples). If no controls are available for a given channel, use
`""` or `NA` for that channel. For example,
`posControls = c("", "(?i)^diap$")` means that channel 1 has no
positive controls, while "diap" is the positive control for channel 2.

The arguments `posControls` and `negControls` are particularly useful in multi-channel data since the controls might be reporter-specific, or after normalizing multi-channel data.

### Value

The function is called for its side-effect.
It returns a character with the full path and name of the report index
file, this is an HTML file which can be read by a web browser.

### Author(s)

Ligia Braz ligia@ebi.ac.uk, Wolfgang Huber huber@ebi.ac.uk

### See Also

`plotPlate`,
`imageScreen`

### Examples

```
 datadir = system.file("KcViabSmall", package = "cellHTS")
 x = readPlateData("Platelist.txt", "KcViabSmall", path=datadir)
 confFile = system.file("KcViabSmall", "Plateconf.txt", package="cellHTS")
 logFile  = system.file("KcViabSmall", "Screenlog.txt", package="cellHTS")
 descripFile  = system.file("KcViabSmall", "Description.txt", package="cellHTS")
 x = configure(x, confFile, logFile, descripFile)
## pCtrls = c("pos") 
## nCtrls = c("neg") 
   # or for safety reasons (not a problem for the current well annotation, however) 
   # pCtrls = c("^pos$") 
   # nCtrls = c("^neg$")
## writeReport(x, posControls=pCtrls, negControls=nCtrls)
 writeReport(x)
 x = normalizePlates(x, normalizationMethod="median",zscore="-")
 x = summarizeReplicates(x, summary="min")
 writeReport(x, force=TRUE, plotPlateArgs = list(), imageScreenArgs=list(zrange=c(-4,4)))
```

---

[Package *cellHTS* version 1.3.23 Index]
