# Supplementary material for: Analysis of cell-based RNAi screens
Source: Genome Biol. 2006 Jul 25;7(7):R66. doi: 10.1186/gb-2006-7-7-r66 (PMC1779553; doi:10.1186/gb-2006-7-7-r66)
Supplement: Additional data file 2 — R package in "Windows binary" format. This file archive also contains the example data. [file gb-2006-7-7-r66-S2.zip › cellHTS/html/writeTab.html]

R: Write the data from a cellHTS object to a tab-delimited file

|  |  |
| --- | --- |
| writeTab {cellHTS} | R Documentation |

## Write the data from a cellHTS object to a tab-delimited file

### Description

Write the data from a `cellHTS` object to a tab-delimited file.

### Usage

```
writeTab(x, ...)
## S3 method for class 'cellHTS':
writeTab(x, file=paste(x$name, "txt", sep="."), ...)
```

### Arguments

|  |  |
| --- | --- |
| `x` | a cellHTS object. |
| `file` | the name of the output file. |
| `...` | ignored. |

### Details

### Value

The name of the file that was written.

### Author(s)

Wolfgang Huber huber@ebi.ac.uk, Ligia Braz ligia@ebi.ac.uk

### Examples

```
 datadir = system.file("KcViabSmall", package = "cellHTS")
 x = readPlateData("Platelist.txt", "KcViabSmall", path=datadir)
 writeTab(x, file=tempfile())
```

---

[Package *cellHTS* version 1.3.23 Index]
